# On the Supervision of a Saturated SIR Epidemic Model with Four Joint Control Actions for a Drastic Reduction in the Infection and the Susceptibility through Time

**DOI:** 10.3390/ijerph19031512

**Published:** 2022-01-28

**Authors:** Manuel De la Sen, Asier Ibeas, Santiago Alonso-Quesada

**Affiliations:** 1 Department of Electricity and Electronics, Institute of Research and Development of Processes, Faculty of Science and Technology, University of the Basque Country, Campus of Leioa, P.O. Box 644 Bilbao, 48940 Leioa, Spain; santiago.alonso@ehu.eus; 2 Department of Telecommunications and Systems Engineering, Universitat Autònoma de Barcelona, UAB, 08193 Barcelona, Spain; Asier.Ibeas@uab.cat

**Keywords:** SIR epidemic model, saturated incidence, contact contagion rate, intervention measures, environment, equilibrium points, vaccination controls

## Abstract

This paper presents and studies a new epidemic SIR (Susceptible–Infectious–Recovered) model with susceptible recruitment and eventual joint vaccination efforts for both newborn and susceptible individuals. Furthermore, saturation effects in the infection incidence terms are eventually assumed for both the infectious and the susceptible subpopulations. The vaccination action on newborn individuals is assumed to be applied to a fraction of them while that on the susceptible general population is of linear feedback type reinforced with impulsive vaccination actions (in practice, very strong and massive vaccination controls) at certain time points, based on information on the current levels of the susceptible subpopulation. Apart from the above vaccination controls, it is also assumed that the average of contagion contacts can be controlled via intervention measures, such as confinements or isolation measures, social distance rules, use of masks, mobility constraints, etc. The main objectives of the paper are the achievement of a strictly decreasing infection for all time periods and that of the susceptible individuals over the initial period if they exceed the disease-free equilibrium value. The monitoring mechanism is the combined activation of intervention measures to reduce the contagion contacts together with the impulsive vaccination to reduce susceptibility. The susceptibility and recovery levels of the disease-free equilibrium point are suitably prefixed by the design of the regular feedback vaccination on the susceptible subpopulation.

## 1. Introduction

Epidemiology is a scientific discipline whose objective is the study and distribution of frequency and determinant factors in the appearance and propagation of infectious diseases, mainly in humans but it is also of interest in plants and animals (both in the wildlife environment and in the farming or agriculture contexts). There is important background literature considering different types of continuous-time and discrete-time epidemic models, some of them considering the incorporation of different vaccination strategies such as constant vaccination, linear feedback vaccination, or impulsive vaccination, the last one implying large vaccination efforts over short periods of time. See, for instance, refs [1,2,3,4,5,6,7,8,9,10,11,12,13,14] and some of the references therein as well as [15,16,17,18,19]. In particular, a stochastic epidemic model that incorporates the effects of media coverage is discussed in [15]. The eventual influence of mixed point and distributed delays on the disease transmission as well as the combined use of regular and impulsive vaccination is focused on and analyzed in detail in [16]. Additionally, the joint effect of constant and impulsive vaccination is discussed in [19] in a Susceptible-Infectious-Susceptible (SIS) epidemic model and in [17] in a proposed epidemic model that incorporates the vaccinated subpopulation into the standard Susceptible-Exposed-Infectious-Recovered (SEIR) epidemic model, while the use of saturated incidence rates in the transmission is considered in [18] and some of references therein. The study of disease evolution in models that include several patches or nodes in integrated networks is also important, since this allows to better fix the influences of the interactions between several geographic areas which can have health systems of different efficiencies, or whose inhabitants have different life conditions or economic power, but that, in fact, interact. See, for instance, refs [20,21,22,23,24,25] and references therein.

The so-called basic reproduction number is a very relevant characteristic of any infectious disease which indicates that the disease-free equilibrium point is locally asymptotically stable (i.e., for small initial disease levels) if it is less than unity. It depends on the parameters of the model, especially, on the disease transmission rate. As the transmission rate increases, the basic reproduction number increases as well. See, for instance, refs [1,2,3,4], and some of the references therein. Physically, such a number gives the number of contagion events of susceptible individuals coming from each infectious one and, mathematically, it establishes that the eigenvalues of the disease-free Jacobian matrix of the linearized model around the disease-free equilibrium point are stable if they are less than one. The introduction of delays in the models, to consider incubation delays, is discussed in several models in [8,9,12] and some of the references therein. Constant and regular vaccination tools, eventually including feedback information on the susceptible subpopulation, have been addressed, for instance, in [4,6,8,10,12,13] and related works. It has been seen that vaccination can improve the basic reproduction number in the sense that it decreases as the feedback vaccination gains increase so that the tolerance to the asymptotic stability of the disease-free equilibrium point increases with respect to the absence of vaccination. Impulsive vaccination has been studied in [13,16,17], and some related works, that analyzed the effects of exerting massive vaccination actions within very short periods of time. In particular, in [16,17] the authors combine impulsive vaccination with regular vaccination in the eventual presence of delays. In [13], fractional calculus tools have been incorporated into the study of epidemic modeling while, in [14,15], a comparative study and relations of dengue and rubella vaccines and the influence of mobility in influenza transmission have been, respectively, performed. See also other related references.

This paper develops a combined study of the influence of potentially joint vaccination efforts on both newborns and the susceptible subpopulation within the general population, taking as a basis a SIR (Susceptible–Infectious–Recovered) epidemic model. This is claimed to be the first design of a proposal for deciding intervention measures through time combined with vaccination laws towards the achievement of a fast extinction of the disease. It is, therefore, considered a SIR model describing the transmission in a certain local environment without interactions with other nodes in a more general patchy environment.

The vaccination effort towards newborns is addressed by a designed fraction coefficient on the susceptible recruitment term. The vaccination effort towards the general susceptible subpopulation is exerted via a combined effort of linear feedback plus impulsive reinforcement actions at certain time instants and it is based on the available information about susceptibility versus time. The impulsive actions are exerted when the susceptible subpopulation starts to increase. The mentioned theoretic impulsive controls can be implemented, in the practice context, over very short periods of time. The three above vaccination controls on newborns and regular and impulsive vaccination on the susceptible subpopulation can be designed in tandem with potential intervention measures such as the use of masks, duty of keeping social distances, mobility constraints, limitation of attendance of people to private and public meetings or spectacles, including eventual closing decrees, measures of quarantines or isolations, etc. Thus, it is possible to exert four control actions under a combined design in order to achieve, as main joint objectives, that:

(a) The infection curve through time is a strictly decreasing function that converges asymptotically to its zero disease-free equilibrium value. *That means, in practice, that the infectious subpopulation decreases continuously to reach the null infection level.*
(b) Possible initial values of the susceptible subpopulation, that exceed their disease-free equilibrium level, are also decreased according to a strictly decreasing profile through the initial time period to levels that are below the disease-free equilibrium. *That means that eventual high levels of the susceptible subpopulation are initially reduced by decreasing them continuously under their expected equilibrium values in the absence of disease*.

*The mathematical concept of impulsive vaccination intuitively means to exert a strong vaccination effort concentrated in a very short time interval to reduce, drastically, an eventual high susceptibility.* If the impulsive vaccination is switched off after a finite time period after the susceptibility level decreases its disease-free value, then the susceptible individuals may evolve to their disease-free equilibrium under the monitoring action of the regular linear feedback control. It turns out that the disease-free equilibrium point values for the susceptible and recovered individuals are partially adjustable with the limit values of the newborns and the susceptible subpopulation regular vaccination limit gains. Thus, both subpopulations might have suitable values in the disease-free steady state or, simpler to address, the fraction of the recovered subpopulation versus the susceptible one might reach an appropriate pre-designed steady-state value. The other two involved controls, namely, the impulsive one on the susceptible subpopulation and the regulation of the infective contacts through intervention measures, are designed as the main tools to achieve the proposed objectives. In particular, the impulsive control on the susceptible subpopulation is designed to achieve an evolution curve that is initially strictly decreasing through time to reach values under the disease-free equilibrium while the intervention measures are designed to achieve a similar effect on the infection curve through time by regulation of the maximum allowed average contagion rates. A set of simulations are performed to emphasize the advantages of the use of vaccination of one or both types compared to the vaccination-free case in the sense that the infection decreases faster under vaccination efforts. The paper is organized as follows. Section 2 describes the proposed epidemic SIR model under the four above mentioned controls as well as the study of the non-negativity and boundedness properties of the trajectory solution. Section 3 is devoted to the study of the fundamental inequality which guarantees that a judicious government of the public intervention measures can achieve an infection curve that is strictly decreasing though time. Section 4 gives the disease-free equilibrium point, depending on the newborn limit vaccination fraction value and the regular vaccination limit gains of the susceptible subpopulation. Section 5 describes the design of the impulsive vaccination and the associated impulsive time periods so that the susceptible subpopulation evolution curve through time is initially a strictly decreasing function evolving to its disease-free equilibrium point value. Section 6 presents and discusses some numerical simulations and, finally, the conclusions end the paper. The proofs of the mathematical results are given in Appendix A in order to maintain easy and direct readability of the main body of the manuscript.

## 2. The Epidemic Model

The following SIR epidemic model, with susceptible subpopulation recruitment and saturated incidence of the susceptible and infectious subpopulations, is considered throughout the paper:

(1) $$\dot{S}\left(t\right)=A-\left(\mu +\frac{\beta \left(t\right)I\left(t\right)}{1+\alpha_{1}S\left(t\right)+\alpha_{2}I\left(t\right)}\right)S\left(t\right)+\rho R\left(t\right)+V\left(t\right)$$

(2) $$\dot{I}\left(t\right)=\frac{\beta \left(t\right)S\left(t\right)I\left(t\right)}{1+\alpha_{1}S\left(t\right)+\alpha_{2}I\left(t\right)}-\left(\mu +\alpha +\gamma \right)I\left(t\right)$$

(3) $$\dot{R}\left(t\right)=\gamma I\left(t\right)-\left(\mu +\rho \right)R\left(t\right)-V\left(t\right)$$

subject to initial conditions $S_{0}=S\left(0\right)\geq 0$, $I_{0}=I\left(0\right)\geq 0$ and $R_{0}=R\left(0\right)\geq 0$, which are assumed throughout the whole paper, where $S\left(t\right)$, $I\left(t\right)$, and $R\left(t\right)$ are the susceptible, infectious, and recovered subpopulations, and $V\left(t\right)$ is the feedback control function, which contains three possible control actions of the general form:

(4) $$V\left(t\right)=-q\left(t\right)A-\left(l\left(t\right)+k\left(t\right)\delta \left(0\right)\right)S\left(t\right)$$

The various parameters in (1)–(4) are the following:

$A\left(\geq 0\right)$: new added individuals per unit of time or susceptible recruitment ratio.
$\mu \left(\geq 0\right)$: mortality rate by natural causes.
$\beta \left(>0\right)$: disease transmission rate.
$\rho \left(\geq 0\right):$ immunity loss rate.
$\alpha \left(\geq 0\right)$: disease mortality rate.
$\gamma \left(\geq 0\right)$: recovery rate.
$\alpha_{1}\left(\geq 0\right)$: susceptible saturation coefficient in the incidence function $\beta SI/\left(1+\alpha_{1}S+\alpha_{2}I\right)$.
$\alpha_{2}\left(\geq 0\right)$: infectious saturation coefficient in the incidence function.
$q\left(\in \left[0\;,\;1\right]\right)$: newborn vaccination effort that vaccinates a fraction of them.
$l\left(\in \left[0\;,\;1\right]\right)$: susceptible feedback vaccination gain to vaccinate a fraction of the susceptible subpopulation via linear feedback.
$q:R_{0+}\to \left[q_{1}\;,\;q_{2}\right]\subset \left[0,1\right]$ is the function defining the instantaneous fraction of vaccinated newborns.
$l:R_{0+}\to \left[l_{1}\;,\;l_{2}\right]\subset \left[0,l_{2}\right]$ is the linear feedback time-varying control gain.
$k:R_{0+}\to \left[0,k_{2}\right)\subset \left[0,\;1\right)$ is the time-varying impulsive control gain, with $\delta \left(.\right)$ being the Dirac distribution.

The interpretation of the last control term, that is the impulsive control, in (4) is that, at certain isolated time instants, vaccination can be applied intensively along a very small (theoretically on an infinitesimal) time interval to largely reinforce the beneficial effects of regular vaccination in fighting against the disease. Consider a nonempty set of zero Lebesgue measures of impulsive time instants Imp=tii=1χ$\left(\chi \leq \infty \right)$ with $t_{i+1}-t_{i}\geq T$ for some arbitrary prescribed T>0. Then, it follows that $k\left(t\right)$ is nonzero if t∈Imp and $k\left(t\right)$ is zero if t∉Imp. In other words, Imp is the support of $k:R_{0+}\to \left[0,k_{2}\right)\subset \left[0,\;1\right)$ so that for any given $\varepsilon \left(<T/2\right)\in R_{+}$, one has:

(5) Sti=Sti+=Sti−−∫ti−εti+εktStδt−tidt=1−ktiSti− ifti∈Imp

(6) St=St− if t∉Imp

and $S\left(t^{+}\right)=\underset{\theta \to 0}{\lim}S\left(t+\theta \right)$, denoted for notation simplicity in the following simply as $S\left(t\right)$, is the right limit of $S\left(t\right)$ at $t=t_{i}$. $S\left(t^{-}\right)=\underset{\theta \to 0}{\lim}S\left(t-\theta \right)$ is its left limit and $S\left(t\right)$ has a bounded discontinuity at t∈Imp, while it is continuous if t∉Imp. As a result, the SIR model (1) under a vaccination control (4) becomes the subsequent closed-loop description:

(7) $$\dot{S}\left(t\right)=A\left(1-q\left(t\right)\right)-\left(\mu +\frac{\beta \left(t\right)I\left(t\right)}{1+\alpha_{1}S\left(t\right)+\alpha_{2}I\left(t\right)}+l\left(t\right)+k\left(t\right)\delta \left(0\right)\right)S\left(t\right)+\rho R\left(t\right)$$

(8) $$\dot{I}\left(t\right)=\frac{\beta \left(t\right)S\left(t\right)I\left(t\right)}{1+\alpha_{1}S\left(t\right)+\alpha_{2}I\left(t\right)}-\left(\mu +\alpha +\gamma \right)I\left(t\right)$$

(9) $$\dot{R}\left(t\right)=\gamma I\left(t\right)-\left(\mu +\rho \right)R\left(t\right)+q\left(t\right)A+\left(l\left(t\right)+k\left(t\right)\delta \left(0\right)\right)S\left(t\right)$$

The solution trajectory of (7)–(9) for any $t\in \left[t_{i}\;,\;t_{i+1}\right]$ and any consecutive ti ,ti+1∈Imp is:

(10) $$S\left(t^{-}\right)=e^{-\int_{t_{i}}^{t}\left(\mu +\frac{\beta \left(\tau \right)I\left(\tau \right)}{1+\alpha_{1}S\left(\tau \right)+\alpha_{2}I\left(\tau \right)}+l\left(\tau \right)\right)d\tau }S\left(t_{i}\right)+\int_{t_{i}}^{t}e^{-\int_{\tau }^{t}\left(\mu +\frac{\beta \left(\xi \right)I\left(\xi \right)}{1+\alpha_{1}S\left(\xi \right)+\alpha_{2}I\left(\xi \right)}+l\left(\xi \right)\right)d\xi }\left(A\left(1-q\left(\tau \right)\right)+\rho R\left(\tau \right)\right)d\tau$$

(11) $$S\left(t\right)=\left(1-k\left(t\right)\right)S\left(t^{-}\right)$$

(12) $$I\left(t\right)=e^{-\int_{t_{i}}^{t}\left(\mu +\alpha +\gamma -\frac{\beta \left(\tau \right)S\left(\tau \right)}{1+\alpha_{1}S\left(\tau \right)+\alpha_{2}I\left(\tau \right)}\right)d\tau }I\left(t_{i}\right)$$

(13) $$R\left(t^{-}\right)=e^{-\left(\mu +\rho \right)\left(t-t_{i}\right)}R\left(t_{i}\right)+\int_{t_{i}}^{t}e^{-\left(\mu +\rho \right)\left(t-\tau \right)}\left(Aq\left(\tau \right)+\gamma I\left(\tau \right)+l\left(\tau \right)S\left(\tau \right)\right)d\tau$$

(14) $$R\left(t\right)=R\left(t^{-}\right)+k\left(t\right)S\left(t^{-}\right)$$

**Remark 1.**  *Note that Equations (1) and (2) have saturated incidences of the susceptible and infectious individuals which are described by the appearing quotient. Note that if the susceptible and infectious subpopulations are at zero, in practice, very low, then there is no effective saturation incidence at all. If any of the two are infinity, in practice, very high, then there is no relevant infection transmission as expected. It can be interpreted that the incidence is modulated by the two coefficients*$\alpha_{1}$*and* $\alpha_{2}$.

Note also that the control law (4) involves two regular vaccination actions, which are the vaccination of newborns, *q(t)A* at time *t*, and the linear feedback vaccination on the susceptible subpopulation of gain, or susceptible fraction to be vaccinated, $l\left(\in \left[0\;,\;1\right]\right)$, plus an impulsive vaccination term also on the susceptible subpopulation. The impulsive vaccination on the susceptible subpopulation also involves feedback information which can reduce drastically the number of susceptible individuals, in practice, in short intervals of time (see (11)). In particular, $k:R_{0+}\to \left[0,k_{2}\right)\subset \left[0,\;1\right)$ is the time-varying impulsive control gain, with $\delta \left(.\right)$ being the Dirac distribution (an instantaneous pulse of zero duration and infinity amplitude). The intuitive interpretation is as follows. If $k\left(t\right)$ is zero, then there is no impulsive vaccination at the time instant *t*. If it is not zero at such time instant, then a fraction of the susceptible subpopulation are vaccinated intensively in a short time interval containing t and the number of susceptible individuals to the right of *t* is reduced by *k*(*t*)*S*(*t*) individuals with respect to its number to the left of value *S*(*t*) at time *t* if *t* is an impulsive time instant; that is, a time instant around which an intensive vaccination is applied. Therefore, an impulsive vaccination is an intensive vaccination concentrated in a very short time interval that can be considered instantaneous from a mathematical point of view. In practice, in a very short time interval, the number of susceptible individuals *S(t)* is reduced by amount *k*(*t*)*S*(*t*). This idea is mathematically reflected in the above Equation (5).

All the controls can be jointly applied, see Equation (4), or the global control can be particularized according to the needs of each particular case, combinations of either pairs or trios of them, or single control actions.

The following result establishes and proves the non-negativity of the state trajectory solution for all time periods under non-negative initial conditions.

**Theorem 1.**  *The following properties hold:*

*(i)*  *The solution trajectory (10)–(14) is non-negative for all time periods for any given impulsive set of time instants irrespective of the vaccination controls.*
*(ii)*  (15) $$\int_{0}^{t}e^{\mu \tau }\left(A+\left(\gamma -\frac{\beta S\left(\tau \right)}{1+\alpha_{1}S\left(\tau \right)+\alpha_{2}I\left(\tau \right)}\right)I\left(\tau \right)\right)d\tau \geq -\eta ;:\forall t\in R_{0+}$$
  *for any non-negative real constant* $\eta \geq N_{0}-I_{0}$*, where* $N_{0}=N\left(0\right)$*is the initial total population.*
*(iii)*  *All the subpopulations are bounded for all time periods for any given finite initial conditions.*
*(iv)* 
(16) $$-e^{-\mu t}\eta \leq \int_{0}^{t}e^{-\mu \left(t-\tau \right)}\left(A+\left(\gamma -\frac{\beta S\left(\tau \right)}{1+\alpha_{1}S\left(\tau \right)+\alpha_{2}I\left(\tau \right)}\right)I\left(\tau \right)\right)<+\infty ;\;\forall t\in R_{0+}$$
$0\leq \underset{t\to \infty }{\lim}\int_{0}^{t}e^{-\mu \left(t-\tau \right)}\left(A+\left(\gamma -\frac{\beta S\left(\tau \right)}{1+\alpha_{1}S\left(\tau \right)+\alpha_{2}I\left(\tau \right)}\right)I\left(\tau \right)\right)<+\infty$

## 3. Control of the Contagion Rate for a Drastic Reduction in the Infection Rate through Time

The subsequent result is a direct consequence of achieving $\dot{I}\left(t\right)<0$ for all nonzero $I\left(t\right)$ at any time from (2) and (8), or equivalently by inspection of the infectious subpopulation trajectory solution (12), and gives drastic conditions for the infection to be strictly decreasing for any given initial conditions:

**Proposition 1.**  $I:R_{0+}\to R_{0+}$*is strictly decreasing if, for each* $t\in R_{0+}$*,*$\beta \left(t\right)<\left(\mu +\alpha +\gamma \right)\left(\alpha_{1}+\frac{1+\alpha_{2}I\left(t\right)}{S\left(t\right)}\right)$*.*

The condition in the above result guarantees that the infection is strictly decreasing for a sufficiently small disease transmission rate related to a combination of some model parameters, irrespective of the vaccination controls. Assuming that $\beta \left(t\right)=c\left(t\right)\vartheta \left(t\right)$, where $c\left(t\right)$ is the average rate of infectious–susceptible contagion contact rate, which depends on population density, social constraints, and intervention measures, such as mandatory use of masks, social distances, quarantines, or confinements; and $\vartheta \left(t\right):R_{0+}\to R_{+}$, which is bounded and positively lower bounded, is a function related to the contributions of other factors such as seasonality. The interpretation of the above equality is not difficult. For instance, it is assumed that at some time instant *t* of the disease evolution, a confinement is announced. Since there are no seasonality contribution effect changes, $\vartheta \left(t\right)$ is invariant around the concrete time instant “*t*”. Thus, the transmission rate can decrease very fast since the infectious contacts are drastically reduced because of the intervention measure immediately after the time instant “*t*”, where the intervention measure is decided, in private meetings or very necessary jobs involving contacts, for instance, according to $\beta \left(t^{+}\right)/\beta \left(t\right)=c\left(t^{+}\right)/c\left(t\right)$. On the other hand, if we compare the disease transmission effects in the same area, with similar population density and similar social constraints, across two different seasons, for instance, at times $t_{1},\;t_{2}$, then the contagious contacts are identical in practice so that the relative transmission rates evolve comparatively for those seasons as $\beta \left(t_{2}\right)/\beta \left(t_{1}\right)=\vartheta \left(t_{2}\right)/\vartheta \left(t_{1}\right)$. In particular, if the pathogen is not seasonal then the transmission rates would be identical, in practice, for those two seasons.

Note that a sufficiently small transmission rate implies a sufficiently small number of average contagion contact rates. Thus, Proposition 1 holds if the average contagion contact rate is small enough to satisfy the subsequent constraint:

$$c\left(t\right)<\frac{\mu +\alpha +\gamma }{\vartheta \left(t\right)}\left(\alpha_{1}+\frac{1+\alpha_{2}I\left(t\right)}{S\left(t\right)}\right);\;\forall t\in R_{0+}$$

It is of interest to study the drastic convergence of the infection to its equilibrium by eventually combining, in the most general case, the four potential involved controls, namely, vaccination of newborns, linear and impulsive vaccination of susceptible individuals, and the control of the transmission rate through some public interventions. For that purpose, it is now assumed that the four controls are piecewise constant with eventual modifications at the impulsive time instants.

The subsequent auxiliary result, to be then used, follows directly from (10)–(14):

**Lemma 1.**  *Assume that*$\beta \left(t\right)=\beta \left(t_{i}\right)=\beta_{i}=c_{i}\vartheta \left(t\right)$*and*$l\left(\tau \right)=l\left(t_{i}\right)=l_{i}$*and*$c\left(t\right)=c\left(t_{i}\right)=c_{i}$*are constant for all*$t\in \left[t_{i}\;,\;t_{i+1}\right)$*,*∀ti,ti+1∈Imp*, where*$c_{i}$*is the average contagion contact rate and*$\vartheta_{i}$*is the contribution to the transmission rate of other factors assumed constant for each interval*$\left[t_{i}\;,\;t_{i+1}\right)$*;*∀ti,ti+1∈Imp*. Then, the solutions (11), (12), and (14) become:*

(17) $$S\left(t\right)=\left(1-k\left(t_{i}\right)\right)S\left(t_{i}^{-}\right)S_{1}\left(t\right)+S_{2}\left(t\right)=S\left(t_{i}\right)S_{1}\left(t\right)+S_{2}\left(t\right)$$

(18) $$I\left(t\right)=e^{-\left(\mu +\alpha +\gamma \right)\left(t-t_{i}\right)+c_{i}\int_{t_{i}}^{t}\left(\frac{\vartheta \left(\tau \right)S\left(\tau \right)}{1+\alpha_{1}S\left(\tau \right)+\alpha_{2}I\left(\tau \right)}\right)d\tau }I\left(t_{i}\right)$$

$$R\left(t\right)=e^{-\left(\mu +\rho \right)\left(t-t_{i}\right)}\left[R\left(t_{i}^{-}\right)+k\left(t_{i}\right)S\left(t_{i}^{-}\right)\right]+\int_{t_{i}}^{t}e^{-\left(\mu +\rho \right)\left(t-\tau \right)}\left(Aq\left(\tau \right)+\gamma I\left(\tau \right)+l_{i}S\left(\tau \right)\right)d\tau$$

$$=e^{-\left(\mu +\rho \right)\left(t-t_{i}\right)}R\left(t_{i}\right)+\int_{t_{i}}^{t}e^{-\left(\mu +\rho \right)\left(t-\tau \right)}\left(Aq\left(\tau \right)+\gamma I\left(\tau \right)+l_{i}S\left(\tau \right)\right)d\tau$$

(19) ∀t∈ti , ti+1, ∀ti , ti+1∈Imp

(20) $$S\left(t_{i+1}\right)=\left(1-k\left(t_{i+1}\right)\right)S\left(t_{i+1}^{-}\right)$$

(21) $$I\left(t_{i+1}\right)=I\left(t_{i+1}^{-}\right)$$

(22) $$R\left(t_{i+1}\right)=R\left(t_{i+1}^{-}\right)+k\left(t_{i+1}\right)S\left(t_{i+1}^{-}\right)$$

*where (17) becomes explicit with the definitions:*

(23) $$S_{1}\left(t\right)=e^{-\left(\mu +l_{i}\right)\left(t-t_{i}\right)}e^{-c_{i}\int_{t_{i}}^{t}\left(\frac{\vartheta \left(\tau \right)}{\left(1+\alpha_{1}S\left(\tau \right)\right)e^{\left(\mu +\alpha +\gamma \right)\left(\tau -t_{i}\right)}e^{-c_{i}\int_{t_{i}}^{\tau }\left(\frac{\vartheta \left(\xi \right)S\left(\xi \right)}{1+\alpha_{1}S\left(\xi \right)+\alpha_{2}I\left(\xi \right)}\right)d\xi }I\left(t_{i}\right)+\alpha_{2}}\right)d\tau }$$

$$S_{2}\left(t\right)=\int_{t_{i}}^{t}e^{-\left(\mu +l_{i}\right)\left(t-\tau \right)}e^{-c_{i}\int_{\tau }^{t}\left(\frac{\vartheta \left(\xi \right)}{\left(1+\alpha_{1}S\left(\xi \right)\right)e^{\left(\mu +\alpha +\gamma \right)\;\left(\xi -t_{i}\right)}e^{-c_{i}\int_{t_{i}}^{\xi }\left(\frac{\vartheta \left(\tau \right)S\left(\tau \right)}{1+\alpha_{1}S\left(\tau \right)+\alpha_{2}I\left(\tau \right)}\right)d\tau }I\left(t_{i}\right)+\alpha_{2}}\right)d\xi }\left(A\left(1-q\left(\tau \right)\right)+\rho^{{}^{{}^{}}}\right.$$

(24) $$\left.\times \left[e^{-\left(\mu +\rho \right)\left(\tau -t_{i}\right)}\left(R\left(t_{i}^{-}\right)+k\left(t_{i}\right)S\left(t_{i}^{-}\right)\right)+\int_{t_{i}}^{\tau }e^{-\left(\mu +\rho \right)\left(\tau -\theta \right)}\left(Aq\left(\theta \right)+\gamma e^{-\left(\mu +\alpha +\gamma \right)\left(\theta -t_{i}\right)}e^{c_{i}\int_{t_{i}}^{\theta }\left(\frac{\vartheta \left(\tau \right)S\left(\tau \right)}{1+\alpha_{1}S\left(\tau \right)+\alpha_{2}I\left(\tau \right)}\right)d\tau }I\left(t_{i}\right)+l_{i}S\left(\theta \right)\right)d\theta \right]\right)d\tau$$;

$\forall t\in \left[t_{i}\;,\;t_{i+1}\right)$, ∀ti , ti+1∈Imp*.*

Now, assume that $\beta \left(t\right)=\beta \left(t_{i}\right)=\beta_{i}=c_{i}\vartheta \left(t\right)$ and $l\left(\tau \right)=l\left(t_{i}\right)=l_{i}$ and $c\left(t\right)=c\left(t_{i}\right)=c_{i}$ are constant for all $t\in \left[t_{i}\;,\;t_{i+1}\right)$, ∀ti, ti+1∈Imp. Therefore, the average contagion contact rate is assumed constant for each inter-impulsive vaccination time period. This assumption is made just to facilitate the subsequent exposition. In particular, note that we can define a strictly increasing set or sequence of time instants $ST={\left\{t_{i}\right\}}_{i=0}^{\chi }$$\left(\chi \leq \infty \right)$ such that Imp⊂ST (the set inclusion being non-proper, in the most general case) such that $c\left(t\right)=c\left(t_{i}\right)=c_{i}$; $\forall t_{i}\in ST$ and to re-address Lemma 1 for such an impulsive set of time instants ST. If ti∈ST∩Imp¯, i.e., $t_{i}$ is in ST but it is not available for impulsive vaccination, then the corresponding impulsive control gain is zeroed (i.e., $k\left(t_{i}\right)=0$) in the corresponding expressions of Lemma 1. In the same way, if $T_{i}=t_{i+1}-t_{i}\leq T_{M}$; $\forall t_{i}\in ST$ and $T_{M}$ is sufficiently small then $\vartheta \left(t\right)$ could be taken as a constant within each time interval $t_{i+1}-t_{i}$ as $\vartheta \left(t\right)=\vartheta \left(t_{i}\right)=\vartheta_{i}$; $\forall t\in \left[t_{i}\;,\;t_{i+1}\right)$; $\forall t_{i}\in ST$ leading to the corresponding associated simplification in the expressions of Lemma 1.

According to Proposition 1, the infection is strictly decreasing if for any consecutive ti,ti+1∈Imp and any $t\in \left[t_{i}\;,\;t_{i+1}\right)$, the average contagion contact rates are small enough to satisfy

(25) ci<fi=fic^i ,t=μ+α+γϑtα1+1+α2ItSt; ∀t∈ti , ti+1, ∀ti , ti+1∈Imp

where

(26) $${\hat{c}}_{i}={\hat{c}}_{i}\left(t\right)=\left\{\begin{array}{c} \left(c_{1},c_{2}\;,\;\cdots ,c_{i}\right);\;t\in \left[0\;,t_{i+1}\right)\\ \left(c_{1},c_{2}\;,\;\cdots ,c_{i},\overset{\nu }{\overset{︷}{\cdots ,c_{i}}}\right);\;t\in \left[t_{i+1}\;,t_{i+\nu }\right)\;for\;2\leq \nu \leq \chi \end{array}\right.$$

for any $i\in \bar{\chi }=\left\{1,2,\cdots ,\chi \right\}$. Note that $c_{i}$ is repeated ν times in (26) since there are no distinct contagion contacts over such time periods. Note that Equation (25) guarantees that the infection function is strictly decreasing. Note that the above constraint is implicit since the average contagion contact rates also appear in its right-hand side (see Lemma 1). Note also that if $c_{i}\equiv 0$ implying and being implied by $I\left(t\right)\equiv 0$, the constraint holds directly as expected from the fact that “no contagion contacts” implies “no infection” under zero initial conditions of the infection and “no infection” implies “no contagion contacts”.

Thus, and for the above argued exposition simplification reasons, we assume in the sequel that ST=Imp, $c\left(t\right)=c\left(t_{i}\right)=c_{i}$, $\vartheta \left(t\right)=\vartheta \left(t_{i}\right)=\vartheta_{i}$, $q\left(t\right)=q\left(t_{i}\right)=q_{i}$ and $l\left(t\right)=l\left(t_{i}\right)=l_{i}$; $\forall t\in \left[t_{i}\;,\;t_{i+1}\right)$, ∀ti, ti+1∈Imp. Thus, the only possibility of $I\left(t\right)/S\left(t\right)$ to be unbounded as time tends to infinity and (25) to hold is that $S\left(t\right)$ tends asymptotically to zero at a faster rate than $I\left(t\right)$. The subsequent result proves that this is not possible as the infection vanishes asymptotically.

**Theorem 2.**  *The following properties hold irrespective of the controls:*

*(i)* 
$0<\frac{\mu +\alpha +\gamma }{\vartheta \left(t\right)}\alpha_{1}\leq f_{i}\left({\hat{c}}_{i}\;,t\right)\leq +\infty$ *;* $\forall t\in R_{0+}$ *,*

$\underset{t\to \infty }{\lim\;\inf}\;f_{i}\left({\hat{c}}_{i}\;,t\right)\geq \alpha_{1}\left(\mu +\alpha +\gamma \right)\left(\underset{t\to \infty }{\lim\;\inf}\left(1/\vartheta \left(t\right)\right)\right)=\lambda_{0}f_{i}\left({\hat{c}}_{i}\;,0\right)>0$ *;* $i\in \bar{\chi }$

*and*

$0<\lambda f_{i}\left({\hat{c}}_{i}\;,0\right)\leq \left(\mu +\alpha +\gamma \right)\left(\frac{\alpha_{1}}{\underset{t\to \infty }{\lim\;\inf}\vartheta \left(t\right)}+\frac{\alpha_{2}}{\underset{t\to \infty }{\lim\;\inf}\left(\vartheta \left(t\right)S\left(t\right)/I\left(t\right)\right)}\right)\leq \underset{t\to \infty }{\lim\;\sup}f_{i}\left({\hat{c}}_{i}\;,t\right)<+\infty$ *;* $i\in \bar{\chi }$

  *in (25) for some*$\lambda_{0},\lambda \in R_{+}$.
*(ii)*  *For any finite arbitrary* $t_{0}\in R_{0+}$ *and* $T\in R_{+}$ *, there is a strictly ordered either finite set or strictly increasing sequence* Imp=ti1χ⊂R+ *(* χ≤+∞ *) of time instants such that the sequence of average contagion contact rates* ${\left\{c_{i}\right\}}_{1}^{\chi }\subset R_{+}$ *, with* $c_{i}\in \left[0,\;{\bar{c}}_{i}\right)$ *;* $i\in \bar{\chi }$ *, satisfies the constraint (25) for some upper-bounding sequence* ${\left\{{\bar{c}}_{i}={\bar{c}}_{i}\left(T_{i}\right)\right\}}_{1}^{\chi }\subset R_{+}$ *where* $T_{i}=t_{i+1}-t_{i}\in {\left[T\;,\;{\bar{T}}_{i}\right)}^{}$ *;* $i\in \bar{\chi }$ *.*

**Remark 2.**  *Related to Theorem 2(ii), note, as it is reflected in its proof, that the admissible range for each* $c_{i}$*(i.e., the allowed levels of infective contagion rates) guaranteeing that (25) holds depends on its previous used values in the inter-impulsive time interval, through (26), and the evolution Equations (17)–(24). However, although (25) is an implicit inequality in* $c_{i}$*, since the last current value of *$c_{i}$*on each interval* $\left[t_{i}\;,\;t_{i+1}\right)$*for* ti ,ti+1∈Imp*appears in both sides, it is solvable. Its solvability arises, according to Theorem 2, since its right-hand side is positively lower-bounded while its left-hand side can be made as close to zero as suitable for a sufficiently small admissible upper bound *${\bar{c}}_{i}$*.*

## 4. Vaccination-Dependent Disease-Free Equilibrium Point under Impulsive Stopping in Finite Time

The following result relies on the existence of a unique disease-free equilibrium point which follows directly by zeroing the time derivatives of (1)–(3) under the assumptions that the vaccination of newborns and the regular feedback vaccination of the susceptible subpopulation have asymptotically convergent gains and that there is only a finite number of vaccination impulsive time instants:

**Theorem 3.**  *Assume that* $k\left(t\right)=0$ *for* $t\geq t_{e}$ *and some arbitrary* $t_{e}<+\infty$ *,* $q\left(t\right)\to q_{df}\in \left[0\;,\;1\right]$ *and* $l\left(t\right)\to l_{df}\in \left[0\;,\;1\right]$ *as* t→∞ *. Then, there exists a unique disease-free equilibrium point which is* $x_{df}={\left(S_{df}\;,\;0\;,R_{df}\right)}^{T}$ *, where:*

(27) $$R_{df}=\frac{\left(l_{df}+\mu q_{df}\right)}{\mu \left(\mu +l_{df}+\rho \right)}A$$

(28) $$S_{df}=\frac{\mu \left(1-q_{df}\right)+\rho }{\mu \left(\mu +l_{df}+\rho \right)}A$$

*and the total population at the disease-free equilibrium point is:*

(29) $$N_{df}=S_{df}+R_{df}=\frac{A}{\mu }$$

**Remark 3.**  *Note from Theorem 3 the important feature that, in the case that any eventual impulsive vaccination control action ends in finite time, either the values of the susceptible and recovered subpopulations, or the ratio of susceptible to recovered subpopulations at the disease-free equilibrium point can be pre-designed by the choices of the gain* $l_{df}$ *and the fraction* $q_{df}$ *which regulate the susceptible vaccination effort and the proportion of vaccinated newborn individuals, respectively. Note, in particular, that such a ratio increases at the disease-free equilibrium as the gain* $l_{df}$ *and the fraction* $q_{df}$ *increase, which follows from the quotient:* $\frac{R_{df}}{S_{df}}=\frac{\left(l_{df}+\mu q_{df}\right)}{\mu \left(1-q_{df}\right)+\rho }$ *.*

## 5. Impulsive Vaccination Control for a Drastic Reduction in the Susceptible Subpopulation through Time

The following result establishes that the monitored impulsive vaccination on a finite set of impulsive time instants can achieve the susceptible population reaching its disease-free equilibrium value in finite time according to a strictly decreasing evolution profile along such a finite time interval. However, this fact does not necessarily imply that this equilibrium value is kept since if the other two subpopulations do not reach their equilibrium values at the same time then the state convergence to the equilibrium has still not been achieved; therefore, the susceptible subpopulation can still evolve through time with time-varying values. The way of monitoring the impulsive vaccination law is to select, as impulsive vaccination time instants, those for which the time derivative of the susceptible subpopulation is positive in the case when the assumption that the initial value of the susceptible subpopulation exceeds its disease-free equilibrium value.

**Theorem 4.**  *Under the constraints of Theorem 3, consider a real function *$\varepsilon :R_{0+}\to R_{+}$*, depending on the initial conditions, which fulfills the constraints*$\varepsilon \left(0\right)\leq S_{0}-S_{df}$*and*$\varepsilon \left(t\right)<1-\frac{S_{df}}{S\left(t^{-}\right)}$*;*∀t∈Imp*with*Imp=t∈R0+ :S˙t>0*. Then,*$S:R_{0+}\to R_{+}$*is strictly decreasing on a finite time interval so that it reaches its equilibrium value the first time in finite time (although this value is not necessarily kept fixed afterwards) by applying vaccination impulses of the form *$k\left(t\right)\delta \left(0\right)$*with* $k\left(t\right)=1-S_{df}/S\left(t^{-}\right)-\varepsilon \left(t\right)$*.*

**Remark 4.**  *As it was mentioned prior to Theorem 4, such a result does not imply that the equilibrium of the susceptible subpopulation has been reached at a finite time. In fact, some of the simulated examples given in Section 6 show that the susceptible curve can be increasing or oscillating after reaching its disease-free equilibrium by the first time and before converging asymptotically to its equilibrium value. Note that, technically, the model of three subpopulations is equivalent to a differential equation of third order for any of the subpopulations so that the equilibrium achievement at some finite time would require that three successive derivatives be jointly zero at such a time instant, or equivalently, that the three first derivatives of the three subpopulations be zero at the same time. Therefore, the normal achievement of the equilibrium is in terms of an asymptotic convergence of the solution as time tends to infinity.*

*As the susceptible subpopulation has finite jumps from its current values to smaller values at the impulsive vaccination time instants, it occurs in parallel that the recovered subpopulation has finite jumps to larger values at the impulsive vaccination time instants of the same modulus as the above ones (see (20) and (22)). This occurs irrespective of the initial conditions of the recovered subpopulation being larger or smaller than that of its disease-free equilibrium value. As a result, the recovered subpopulation evolution through time cannot be decreasing everywhere, irrespective of whether the initial conditions are larger or smaller than the disease-free equilibrium value.*

**Remark 5.**  *Note from Theorems 2 (or from Proposition 1), 3, and 4, and Remark 4 that the infectious and the susceptible subpopulations’ evolution through time can be strictly decreasing everywhere at the first time interval if the contagion contact rate and the impulsive vaccination are monitored “ad hoc” for that purpose, provided that the susceptible subpopulations’ initial conditions exceed its disease-free equilibrium value. The susceptible subpopulation can be strictly decreasing until levels are below its disease-free equilibrium value along such initial finite time period. The infectious subpopulation can continue to be strictly decreasing afterwards, while the susceptible subpopulation increases from values under its equilibrium level to asymptotically reach its disease-free equilibrium value.*

The combined convergence of the infectious and the susceptible subpopulations to their respective disease-free equilibrium values based on the strategies and results of Theorem 2/Proposition 1 and Theorems 3 and 4 is addressed in the next result:

**Theorem 5.**  *The following properties hold:*

*(i) Assume that the hypotheses of Proposition 1, or those of Theorems 2, 3, and 4 all hold. Assume also that*$R_{0}<R_{df}$*and*$0<I_{0}<\frac{A-\mu \left(S_{0}+R_{0}\right)}{\alpha +\mu }$*. Then,*$I:R_{0+}\to R_{+}$*is strictly decreasing with zero limit,*$S:R_{0+}\to R_{+}$*is strictly decreasing on a finite time interval to reach its equilibrium value*$S_{df}$*by the first time instant while*$R:R_{0+}\to R_{+}$*is strictly increasing on the same finite time interval. Additionally,*$S,I\;,R:R_{0+}\to R_{+}$*asymptotically reach their disease-free equilibrium values*$S_{df}$*,*0*, and*$R_{df}$.

*(ii) Assume that the hypotheses of Proposition 1, or those of Theorems 2, 3, and 4 all hold. If* $I_{0}\geq \frac{A-\mu \left(S_{0}+R_{0}\right)}{\alpha +\mu }$ *, then either* $R\left(t\right)\leq R_{df}$ *for all finite* $t\in R_{0+}$ *and* $R\left(t\right)\to R_{df}$ *or* $R_{0}>R_{df}$ *and* $R\left(t\right)$ *converge to* $R_{df}$ *.*

**Remark 6.**  *A judicious way of guaranteeing that both the infectious and the susceptible subpopulations evolve according to a strictly decreasing function is the following:*

- *To select the fraction of vaccinated newborns and the regular feedback control gain at the disease-free equilibrium point to fix the suitable disease-free equilibrium values of the susceptible and recovered subpopulations. See Theorem 3, Equations (27) and (28).*
- *Apply the necessary public intervention measures for monitoring the contagion contact rate between individuals to keep the contagion rate within admissible ranges which guarantee that the infectious subpopulation functions’ evolution with time is strictly decreasing. See Theorem 2 and Equations (25) and (26). For this purpose, the control gains for newborn vaccination and for vaccination of the susceptible individuals are designed to converge to the suitable values to prefix the disease-free equilibrium point.*
- *Apply impulsive vaccination control with its gains and associated impulsive time instants is designed so that the susceptible subpopulation evolution function with time is strictly decreasing and converges to its disease-free equilibrium point prefixed value. See Theorem 4.*

## 6. Simulation Examples

This section contains some numerical simulation examples regarding the theoretical results introduced in the previous sections. Thus, consider the SIR model given by (1)–(3) with parameters given in Table 1 and initial conditions S(0) = 799, I(0) = 201, R(0) = 0.

**Figure 1:**
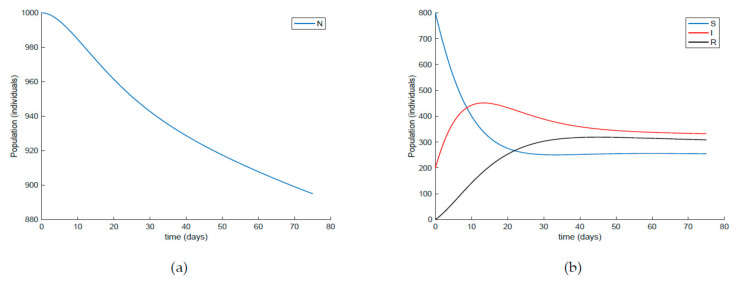
(**a**) Evolution of the total population, and (**b**) each subpopulation according to model (1)–(3) with parameter values given by Table 1 and in the absence of external actions.

Figure 1 displays the evolution of the three subpopulations along with the total population. It is observed in Figure 1 that the trajectory is non-negative and bounded for all time periods as is proved in Theorem 1. Moreover, the total population is bounded and decreases through time. In addition, it is shown in Figure 1 that the infectious individuals do not vanish over time and there are always a few of them within the population. Four counteracting measures can be adopted to make the infectious individuals vanish asymptotically. The effects of all these measures are discussed in this section using numerical examples.

**Figure 2:**
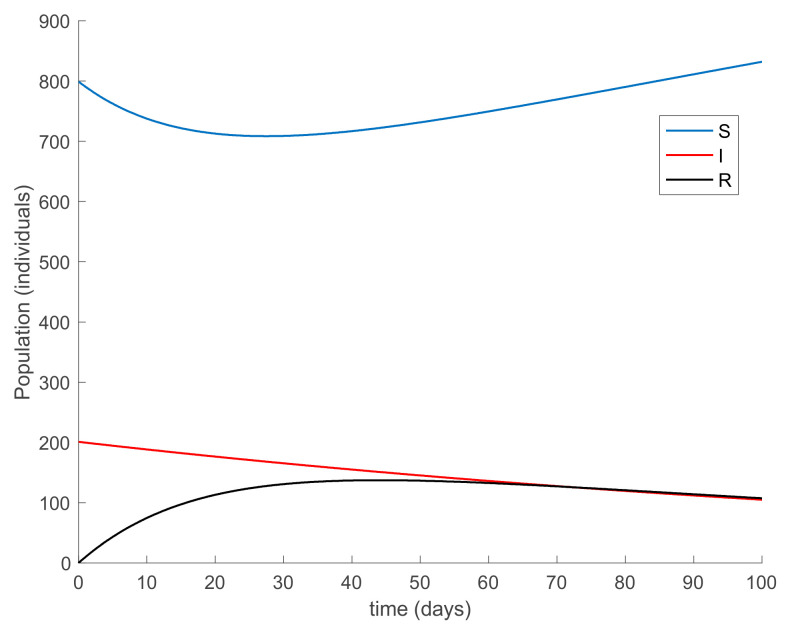
Evolution of the SIR model when *λ* = 0.9 is used in Equation (30) to determine the value of *β*.

**Figure 3:**
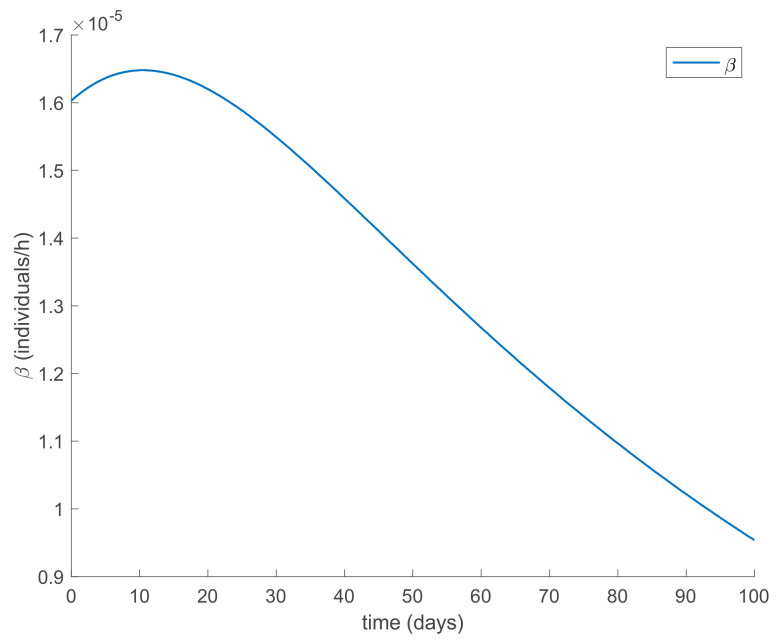
Evolution of β when it is selected using Equation (30) with *λ* = 0.9.

**Figure 4:**
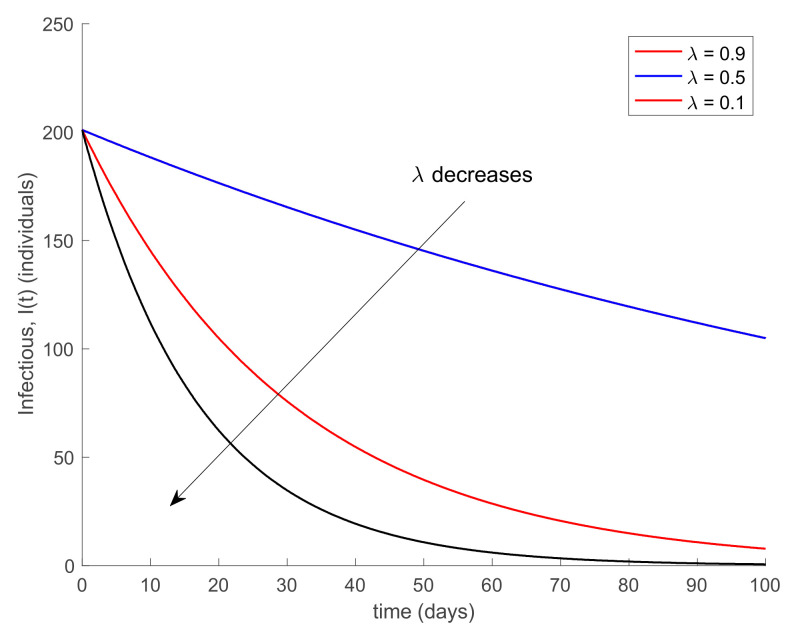
Evolution of the infectious subpopulation for different values of *λ* and *β* given by Equation (30).

**Figure 5:**
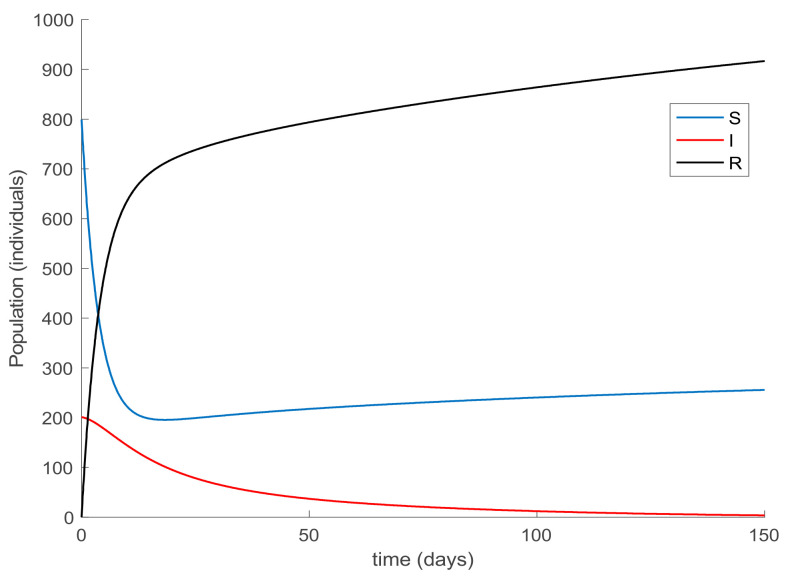
Evolution of all of the subpopulations when a constant vaccination of 20% of the susceptible subpopulation is added to the control of the contagion rate. The value of *β* is given by Figure 3.

**Figure 6:**
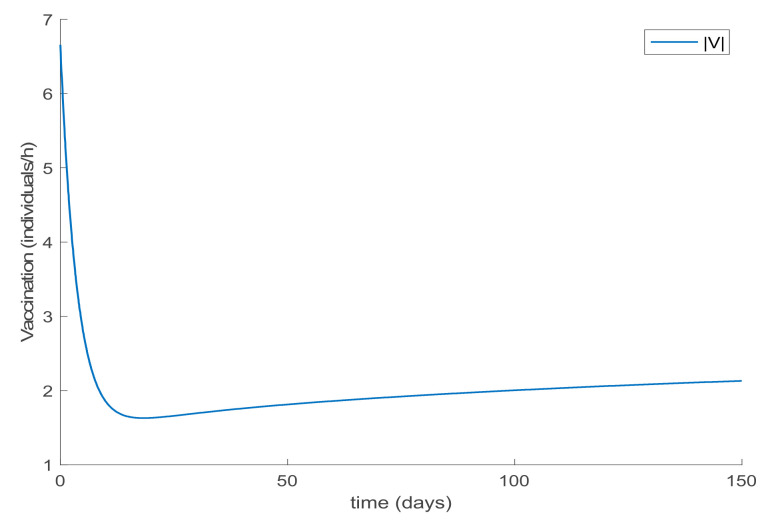
Vaccination applied when a constant term of the susceptible individuals is vaccinated (20%) and β is given by Figure 3.

The first control action is introduced in Section 3 as the control of the contagion rate, captured by β. Thus, the value of β is changed through time by forcing lockdowns, minimum separation distances between individuals, the use of face masks, etc. In this way, the value of β is expressed as:

(30) $$\beta \left(t\right)=\lambda \left(\mu +\alpha +\gamma \right)\left(\alpha_{1}+\frac{1+\alpha_{2}I\left(t\right)}{S\left(t\right)}\right)$$

with $\lambda \in \left[0,1\right)$ so that (25) holds over all time periods. Figure 2 displays the evolution of the SIR model when *λ* = 0.9 and Figure 3 shows the value of β that resulted from Equation (30). In addition, Figure 4 depicts the evolution of the infectious individuals for different values of *λ*. It is deduced from Figure 2 and Figure 4 that the population of infectious individuals is strictly decreasing with time, as Proposition 1 claims. In addition, the selection of the contagion rate through the adoption of social measures can make the number infectious individuals converge to zero, the rate of which is faster as the measures taken are stronger, resulting in a smaller contagion rate. The social measures could be relaxed, or the infectious population could vanish in a faster way if other measures are adopted along with the control of the contagion rate. Three additional control measures are considered in this work on top of the control of β, namely, the vaccination of newborns/newcomers, the constant vaccination of susceptible individuals, and the impulsive vaccination of susceptible individuals. Therefore, the value of β depicted in Figure 3 is now considered as the one parameterizing models (1)–(3) while a constant vaccination of 20% of the susceptible subpopulation is also added. Figure 5 shows the evolution of the subpopulations in this case. It is shown that the application of vaccination improves the vanishing rate of the infectious individuals for the same value of *β*. Thus, the infectious subpopulation asymptotically tends to zero due to the combined action of contagion rate control and constant vaccination. Figure 6 depicts the vaccination function applied, shown in absolute values. As it would have been expected, the vaccination effort is larger at the beginning of the outbreak and decreases over time.

**Figure 7:**
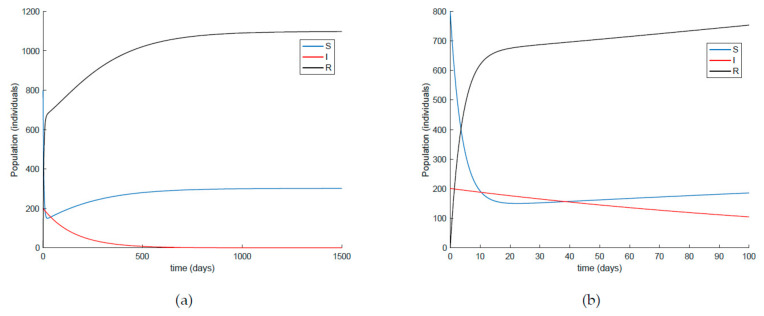
(**a**) Evolution of the system when β  is fixed through Equation (30) (λ=0.9) and a constant vaccination term of 20% of the susceptible individuals is also employed. (**b**) Detail of the simulation for the first 100 days.

**Figure 8:**
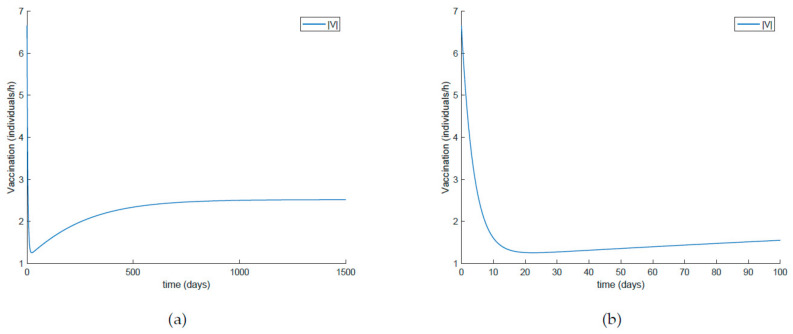
(**a**) Vaccination function when β is fixed through Equation (30) (λ=0.9) and a constant vaccination term of 20% of the susceptible individuals is also employed. (**b**) Detail of the simulation for the first 100 days.

**Figure 9:**
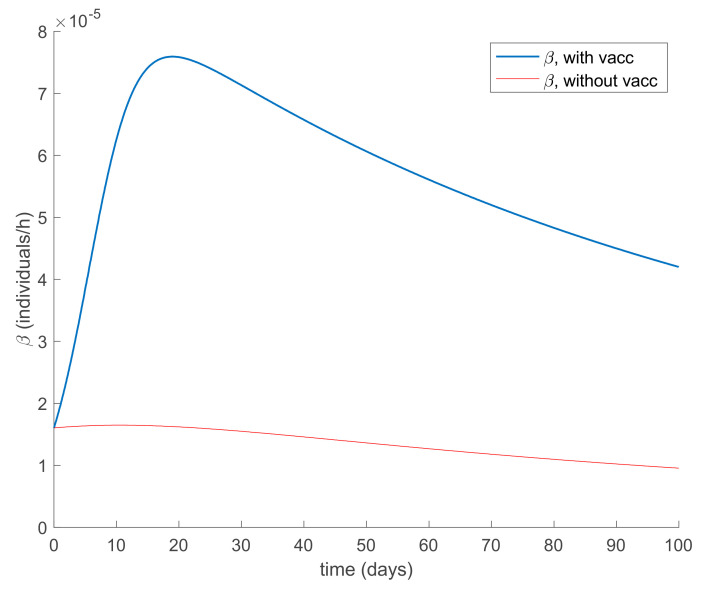
Different values of *β* calculated through (30) corresponding to the application of constant vaccination and the absence of it.

On the other hand, we now fix the value of *β* according to (30) with *λ* = 0.9 and a constant vaccination of 20% of the susceptible individuals is also added. In this way, both control actions are applied simultaneously. Figure 7 shows the response of the system in this case. Figure 8 depicts the corresponding vaccination function. Moreover, Figure 9 displays the values of *β* when Equation (30) is employed and in one case vaccination is not used while in the other case it is used. It can be deduced from Figure 9 that when vaccination is applied in addition to the control of contagion rate, the value of *β* for the same infectious decreasing rate is allowed to be larger. This fact means that vaccination allows relaxing of the adopted social measures while controlling the infection spread. It can also be observed in Figure 7 that the system reaches a disease-free equilibrium point given by:

$$R_{df}=\frac{\left(l_{df}+\mu q_{df}\right)}{\mu \left(\mu +l_{df}+\rho \right)}A=1098$$

$$S_{df}=\frac{\mu \left(1-q_{df}\right)+\rho }{\mu \left(\mu +l_{df}+\rho \right)}A=301.96$$

in accordance with the numerical values provided by the simulation and given by *R_df_* = 1097.3 and *S_df_* = 301.78 after 1500 days of simulation, as Theorem 3 predicts.

**Figure 10:**
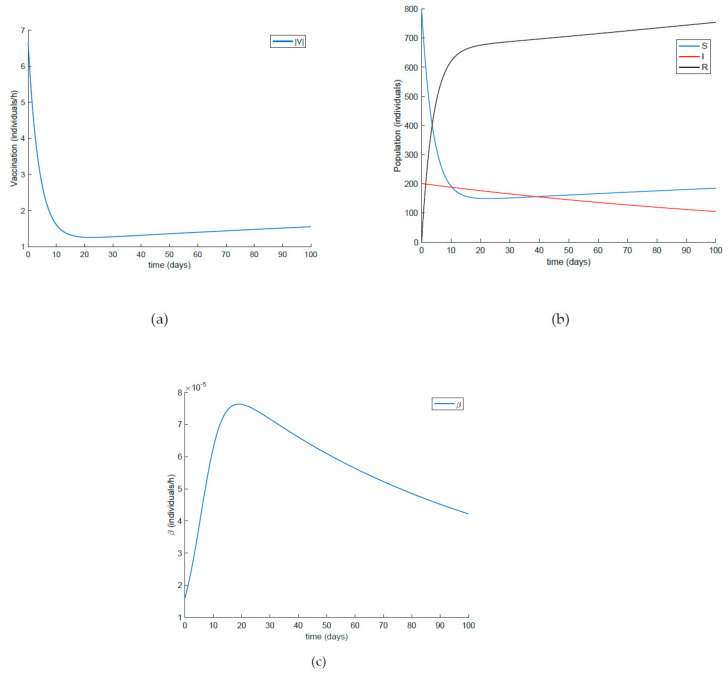
Results obtained when vaccination of the newcomers is added to the control of the contagion rate and the constant vaccination to the susceptible subpopulation, (**a**) vaccination control, (**b**) populations, (**c**) transmission rate.

The constant vaccination of the susceptible individuals may be also combined with the vaccination of the newcomers/newborns. Thus, the value of *β* is fixed through (30) with *λ* = 0.9, the susceptible individuals are vaccinated at a rate of 20%, while the newborns are vaccinated at a rate of 70%. The results are shown in Figure 10 where the evolution of all the subpopulations, the vaccination function, and *β* are shown. Since the value of *A* is only of seven individuals per day (for a total population of 1000 individuals), the effect of newborn vaccination does not have much of an influence on the spread of the infection. Thus, only when the *A* term is large enough would this vaccination impact the system’s evolution. As a consequence, this term is only recommended to be applied when the inflow of newborns is large.

**Figure 11:**
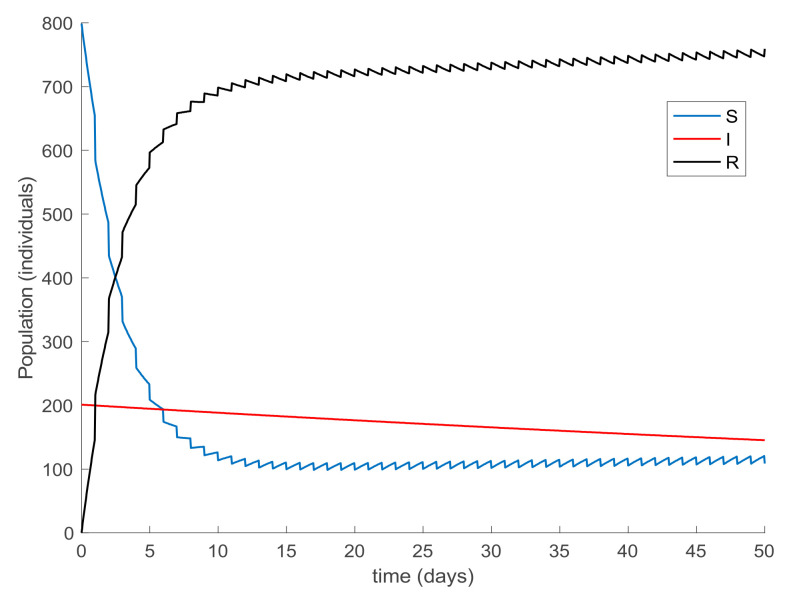
Evolution of all subpopulations when an impulsive vaccination of the susceptible individuals is added to the previous actions.

**Figure 12:**
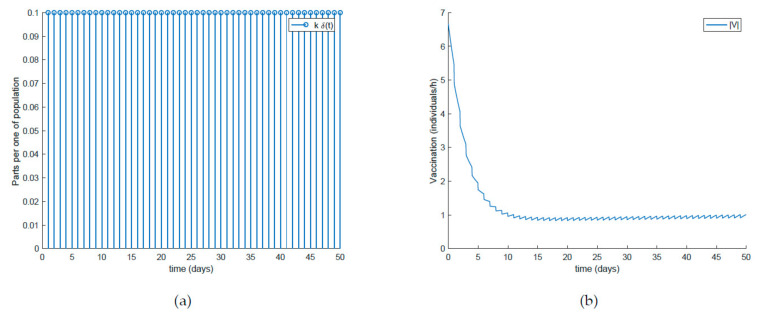
(**a**) Impulsive vaccination (everyday) and (**b**) total feedback vaccination applied.

**Figure 13:**
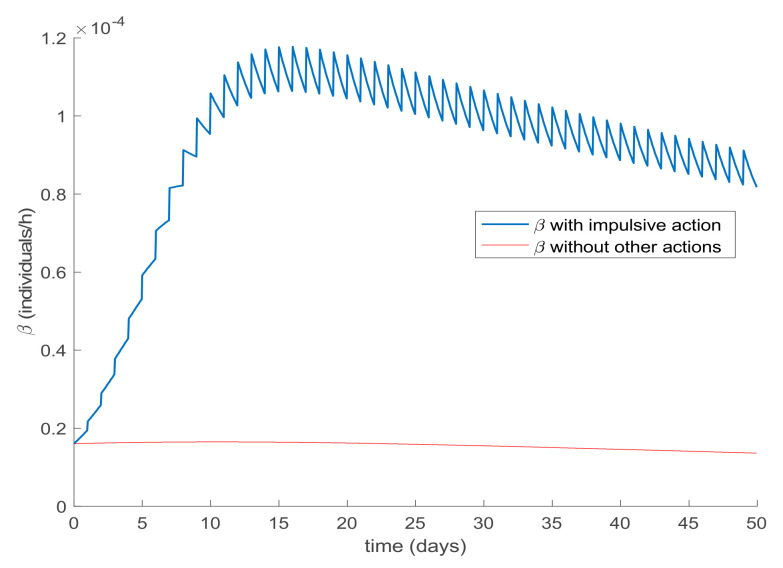
Value of *β* obtained through (30) when only the contagion rate is applied and when all control actions are employed.

Finally, the effect of impulsive vaccination is discussed. In addition to the previous actions (the value of *β* is fixed through (30) with *λ* = 0.9, the susceptible individuals are vaccinated at a rate of 20%, and the newborns are vaccinated at a rate of 70%), an impulsive vaccination is applied with different periodicities. Thus, initially the impulsive vaccination is applied to 10% of the susceptible subpopulation during 1h every day. This means that the total amount of vaccines is applied in the relatively short period of time of 1 h every day. The results are displayed in Figure 11, while the vaccination function is shown in Figure 12. It is observed by comparing Figure 7 and Figure 11 that the susceptible subpopulation decreases faster when the impulsive vaccination is added while the immune subpopulation increases faster. The value of *β* corresponding to this situation is also depicted in Figure 13, in comparison with the value obtained previously when only the contagion rate control was implemented (Figure 3). The addition of this new type of vaccination potentially reduces the severity of social restrictions as Figure 13 shows for the *β* function. Consequently, as vaccination is available and applied, the social restrictions may be softened while having the epidemic spread under control.

**Figure 14:**
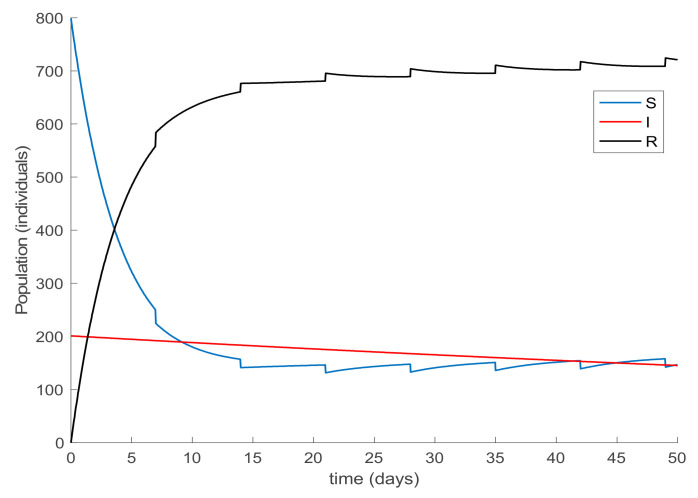
Evolution of all the subpopulations when an impulsive vaccination of susceptible individuals one day per week is added to the previous actions.

**Figure 15:**
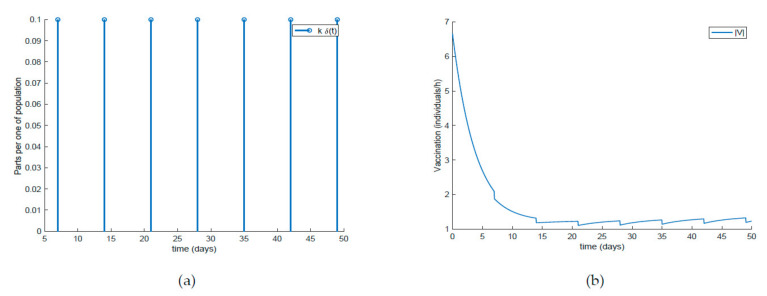
(**a**) Impulsive vaccination (once per week) and (**b**) total feedback vaccination applied.

**Figure 16:**
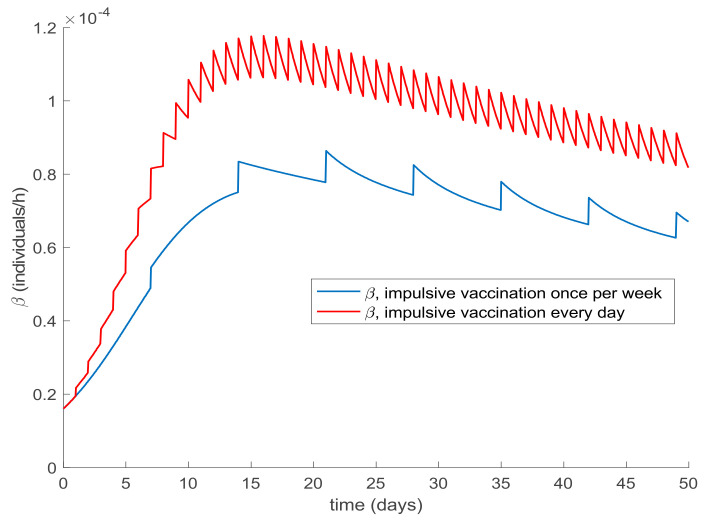
Value of *β* obtained through (30) when the impulsive vaccination is applied once per week and every day.

Figure 14 shows what happens when the impulsive vaccination is applied during 1 h one day per week. As it could have been expected, the susceptible decrease and immune increase are slower than in the previous application schema. Furthermore, the effort of impulsive vaccination is reduced in comparison to the previous case as can be observed in Figure 15, where the vaccination functions are depicted. Figure 16 displays the value of *β* obtained via (30) with *λ* = 0.9 when the impulsive vaccination is applied every day and only once per week. As the vaccination effort is larger, the value of *β* is also larger, while having controlled the disease. This fact means that social restrictions are less important as vaccination rate is stronger.

**Figure 17:**
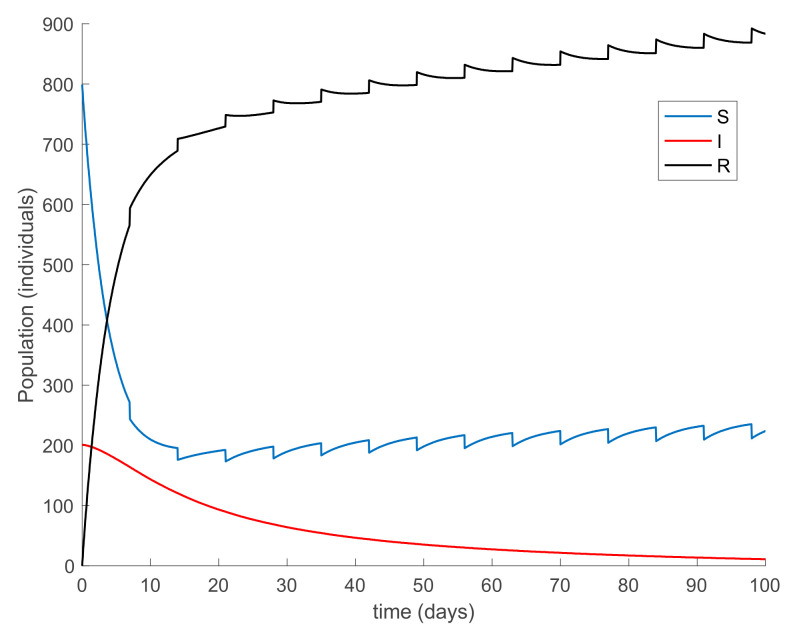
Evolution of all the subpopulations when a variable impulsive vaccination of the susceptible individuals one day per week is added to the previous actions.

**Figure 18:**
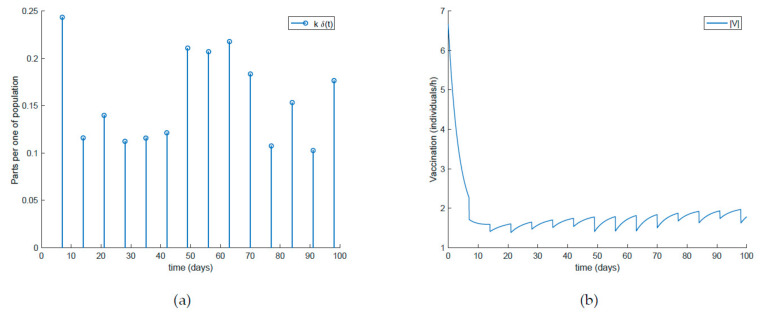
(**a**) Impulsive vaccination (once per week with time-varying amplitude) and (**b**) total feedback vaccination applied.

**Figure 19:**
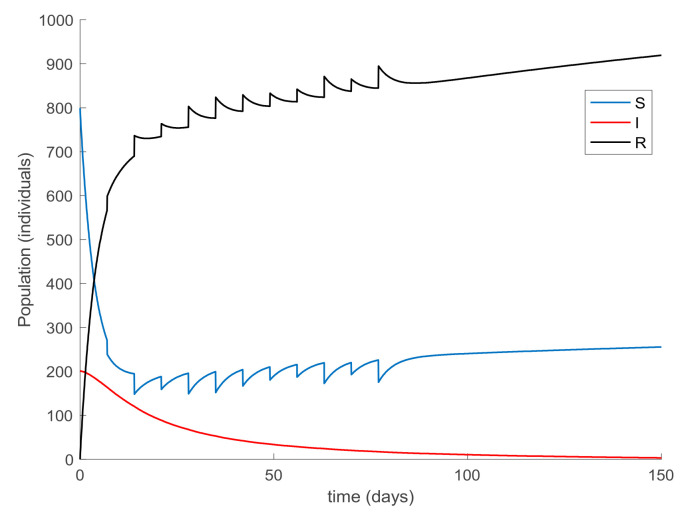
Evolution of all the subpopulations when a variable and limited-in-time impulsive vaccination of the susceptible individuals one day per week is added to the previous actions.

**Figure 20:**
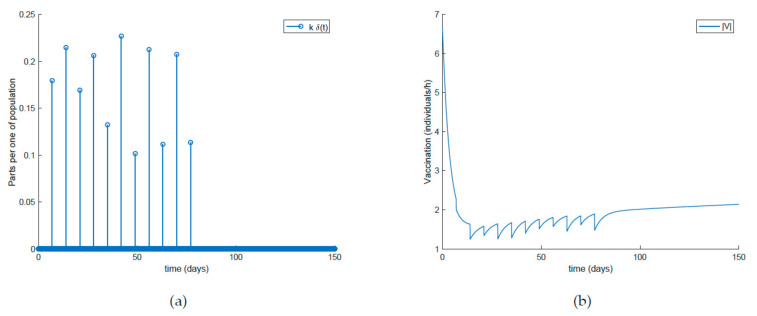
(**a**) Impulsive vaccination (once per week limited-in-time with time-varying amplitude) and (**b**) total feedback vaccination applied.

Figure 17 shows the dynamics of the SIR model when the amplitude of the impulsive vaccination is not constant over time, that is, it is different for each impulse time. Additionally, the corresponding vaccination is displayed in Figure 18. Finally, Figure 19 and Figure 20 depict the simulation results when the impulsive vaccination stops in finite time. It is observed in Figure 19 that when the impulsive action finishes, the evolution of the susceptible and immune individuals is smooth and they tend toward the disease-free equilibrium point (implying that the infectious subpopulation vanishes asymptotically). Overall, it is shown through simulation examples that the considered actions are effective to control the infection spread, and the fact that vaccination plays a crucial role for relaxing social restrictions while controlling the epidemic spread.

**Figure 21:**
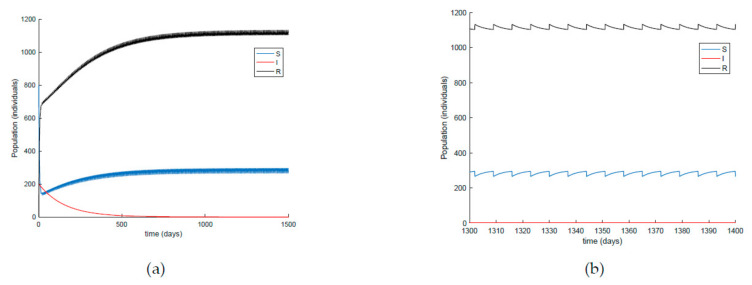
(**a**) Extended simulation time in Figure 14 and (**b**) zoomed image of the final time period for the susceptible and recovered individuals.

**Figure 22:**
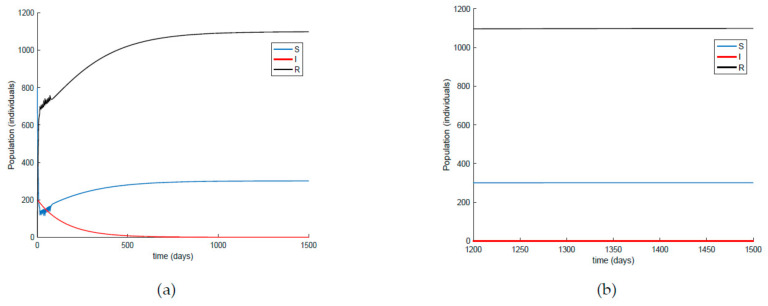
(**a**) Extended simulation time in Figure 19 and (**b**) zoomed image of the final time period for the susceptible and recovered individuals with limited-in-time impulsive vaccination effort.

On the other hand, Figure 21 displays an extended simulation for a longer evaluation time than that of Figure 14, in particular over 1500 days, and details of the susceptible and recovered individuals over the final evaluation time. It is corroborated in Figure 21 that the infection curve versus time is strictly decreasing while the susceptible subpopulation firstly decays with a strictly decreasing profile to levels under its disease-free equilibrium value while afterwards increases to values that oscillate around its equilibrium position. The experiment is then modified in Figure 22 with the impulsive vaccination being switched off after a finite time once the susceptibility has fallen below its disease-free equilibrium value. Note that the susceptible and the recovered subpopulations converge to their equilibrium values.

## 7. Conclusions

This paper considered an SIR epidemic model with susceptible subpopulation recruitment which incorporates also vaccination of a proportion of newborns, acting on the susceptible subpopulation recruitment, and vaccination of the remaining susceptible individuals. The second mentioned vaccination strategy incorporates updated regular linear feedback information of the susceptible subpopulation plus impulsive vaccination at certain impulsive time instants, involving also feedback information. Both vaccination controls can be planned based on the use of time-varying vaccination gains. Furthermore, the appropriate public intervention orders, of mandatory application, which regulate measures such as confinement, isolation, mandatory use of masks, social distance rules, mobility constraints, reduction in number attendees to meetings, spectacles and leisure, etc., are considered to govern the contagion contact rate. As a result, a proper design of the impulsive vaccination gains at the impulsive time instants combined with a proper application of the intervention measures can achieve a joint drastic reduction through time in the susceptible and infectious subpopulations, which could also converge asymptotically to their disease-free equilibrium values. Such disease-free equilibrium values, or their ratio, might be prefixed by the judicious choice of the limits of the regular vaccination gains and that of the fraction of vaccinated newborns. Performed numerical simulations corroborated the theoretical results.

## Figures and Tables

**Table 1:** Parameters for the models (1)–(3) employed in the simulation examples.

Parameter	Value (Individuals/Day)
A	7
β	0.002
$\alpha_{1}$	0.002
$\alpha_{2}$	0.5
ρ	0.05
γ	0.05
μ	0.005
α	0.01

## Data Availability

Not applicable.

## Appendix A. Mathematical Proofs

**Proof** **of** **Theorem** **1.** Note from (8) that, since $I\left(0\right)=I_{0}\geq 0$ then $I\left(t\right)=e^{-\int_{0}^{t}\left(\mu +\alpha +\gamma -\frac{\beta \left(\tau \right)S\left(\tau \right)}{1+\alpha_{1}S\left(\tau \right)+\alpha_{2}I\left(\tau \right)}\right)d\tau }I_{0}\geq 0$; $\forall t\in R_{0+}$. First, note from the above equation that the infectious subpopulation can only be zero at the initial time instant or at infinity but not at any non-zero finite time instant. Now, consider three possible cases considering the eventual zeroing of the susceptible and/or recovered subpopulations at a finite time instant:

1. *The susceptible and recovered subpopulations are jointly zero at some finite time instant*. Assume that for some $t\in R_{0+}$, both $S\left(t\right)=R\left(t\right)=0$ and $S\left(\tau \right)\geq 0$ and $R\left(\tau \right)\geq 0$ for $\tau \in \left[0,t\right)$. Then, $\dot{R}\left(t^{-}\right)=\gamma I\left(t\right)+q\left(t\right)A\geq 0$ so that $R\left(t^{-}\right)\geq 0$. Additionally, since $R\left(t\right)=0$, $\dot{S}\left(t^{-}\right)=A\left(1-q\left(t\right)\right)+\rho R\left(t\right)=A\left(1-q\left(t\right)\right)\geq 0$, then $S\left(t^{-}\right)\geq 0$ so that $S\left(t\right)=\left(1-k\left(t\right)\right)S\left(t^{-}\right)\geq 0$ and then $S\left(\tau \right)\geq 0$ for $\tau \in \left[0,t\right]$ and also $R\left(t\right)=R\left(t^{-}\right)+k\left(t\right)S\left(t^{-}\right)\geq 0$ so that $R\left(\tau \right)\geq 0$ for all $\tau \in \left[0,t\right]$. Thus, $S\left(t\right)\geq 0$ for all $t\in R_{0+}$ since $S_{0}\geq 0$.
2. *The susceptible subpopulation is zero at some finite time instant*. Assume that for some $t\in R_{0+}$, $S\left(t\right)=0$, $S\left(\tau \right)\geq 0$ for $\tau \in \left[0,\;t\right)$ and $R\left(\tau \right)\geq 0$ for $\tau \in \left[0,t\right]$. Then, again $\dot{S}\left(t^{-}\right)=A\left(1-q\left(t\right)\right)+\rho R\left(t\right)\geq 0$, which leads, in the same way as above, to $S\left(\tau \right)\geq 0$ for $\tau \in \left[0,t\right]$. As a result, $S\left(t\right)\geq 0$ for all $t\in R_{0+}$ since $S_{0}\geq 0$.
3. *The recovered subpopulation is zero at some finite time instant*. Assume that for some $t\in R_{0+}$, $R\left(t\right)=0$ and $R\left(\tau \right)\geq 0$ and $S\left(\tau \right)\geq 0$ for $\tau \in \left[0,t\right]$. Then, $\dot{R}\left(t^{-}\right)=\gamma I\left(t\right)+q\left(t\right)A+\left[l\left(t\right)+k\left(t\right)\delta \left(0\right)\right]S\left(t\right)\geq 0$ which leads, as in Case 1, to $R\left(\tau \right)\geq 0$ for $\tau \in \left[0,t\right]$. As a result, $R\left(t\right)\geq 0$ for all $t\in R_{0+}$ since $R_{0}\geq 0$.

Property (i) has been proved. To prove Property (ii), note that by summing-up (7) and (9), one gets:

(A1) $$\dot{S}\left(t\right)+\dot{R}\left(t\right)=A-\mu \left(S\left(t\right)+R\left(t\right)\right)+\left(\gamma -\frac{\beta S\left(t\right)}{1+\alpha_{1}S\left(t\right)+\alpha_{2}I\left(t\right)}\right)I\left(t\right)$$

whose solution is

(A2) $$S\left(t\right)+R\left(t\right)=e^{-\mu t}\left[S_{0}+R_{0}+\int_{0}^{t}e^{\mu \tau }\left(A+\left(\gamma -\frac{\beta S\left(\tau \right)}{1+\alpha_{1}S\left(\tau \right)+\alpha_{2}I\left(\tau \right)}\right)I\left(\tau \right)\right)d\tau \right]\geq 0;\forall t\in R_{0+}$$

with the non-negativity of (A2) being a direct consequence of Property (i). Property (ii) follows directly from (A2) by using the identity $N_{0}-I_{0}=S_{0}+R_{0}$. To prove Property (iii), summing the three Equations (7)–(9) to yield the total population $N\left(t\right)=S\left(t\right)+I\left(t\right)+R\left(t\right)$ satisfies the differential equation:

$$\dot{N}\left(t\right)=-\mu N\left(t\right)+A-\alpha I\left(t\right)\leq -\mu N\left(t\right)+A;\;N\left(0\right)=N_{0}$$

then

$$N\left(t\right)\leq e^{-\mu t}N_{0}+A\int_{0}^{t}e^{-\mu \left(t-\tau \right)}d\tau \leq N_{0}+\frac{A}{\mu };\;\underset{t\to \infty }{\lim\;sup\;N}\left(t\right)\leq \frac{A}{\mu }$$

Since all the subpopulations are non-negative for all time periods from Property (i) and the total population is bounded for all time periods from the above discussion, then all the subpopulations are bounded for all time periods. Property (iii) has been proved. Property (iv) follows from (16) (see also Property (ii)). □

**Proof** **of** **Theorem** **2.** The property $\frac{\mu +\alpha +\gamma }{\vartheta \left(0\right)}\alpha_{1}\leq f\left({\hat{c}}_{i}\;,0\right)\leq +\infty$; $i\in \bar{\chi }$ follows directly from (25) since $\vartheta \left(t\right)$ is everywhere positively lower bounded and finitely upper bounded by noting also that $f\left({\hat{c}}_{i}\;,0\right)$ is infinity if $S_{0}=0$. The subsequent constraints hold:

$$\underset{t\to \infty }{\lim\;\inf}\;f_{i}\left({\hat{c}}_{i}\;,t\right)\geq \alpha_{1}\left(\mu +\alpha +\gamma \right)\left(\underset{t\to \infty }{\lim\;\inf}\left(1/\vartheta \left(t\right)\right)\right)>0$$

$$\left(\mu +\alpha +\gamma \right)\left(\frac{\alpha_{1}}{\underset{t\to \infty }{\lim\;\inf}\vartheta \left(t\right)}+\frac{\alpha_{2}}{\underset{t\to \infty }{\lim\;\inf}\left(\vartheta \left(t\right)S\left(t\right)/I\left(t\right)\right)}\right)\leq \underset{t\to \infty }{\lim\;\sup}\;f_{i}\left({\hat{c}}_{i\;,}t\right)$$

for all $i\in \bar{\chi }$ follow directly from (25) and the positivity and boundedness of $\vartheta \left(t\right)$ on $R_{0+}$. It has to be proved that $\underset{t\to \infty }{\lim\;\sup}\;f_{i}\left({\hat{c}}_{i}\;,t\right)<+\infty$; any $i\in \bar{\chi }$, which implies $\underset{t\to \infty }{\lim\;\sup}\left(I\left(t\right)/S\left(t\right)\right)<+\infty$, as a result. Note that $f_{i}\left({\hat{c}}_{i}\;,t\right)\to +\infty$; for any $i\in \bar{\chi }$ as t→∞, which contradicts $\underset{t\to \infty }{\lim\;\sup}\;f_{i}\left({\hat{c}}_{i}\;,t\right)<+\infty$; for any $i\in \bar{\chi }$, this occurs only if $\frac{I\left(t\right)}{S\left(t\right)}\to +\infty$ as t→∞ since $+\infty >\vartheta \left(t\right)>0$; $\forall t\in R_{0+}$. We now prove that $\underset{t\to \infty }{\lim\;\sup}\frac{I\left(t\right)}{S\left(t\right)}<+\infty$. Consider two possible cases, namely:

(a) If $I\left(0\right)=0$ then $I\left(t\right)$ is identically zero for all time periods and then $I\left(t\right)/S\left(t\right)$ is also zero and then bounded for all time periods. Then, $\underset{t\to \infty }{\lim\;\sup}\;f\left({\hat{c}}_{i}\;,t\right)<+\infty$.
(b) Assume that $I\left(0\right)>0$ and that (25) holds then $I:R_{0+}\to R_{0+}$ is bounded for all time periods since it is strictly decreasing from Proposition 1. Assume, on the contrary, that $I\left(t\right)/S\left(t\right)$ is unbounded as time tends to infinity. Since $I\left(t\right)$ converges to zero as time tends to infinity, then $S\left(t\right)$ also converges asymptotically to zero at a faster rate than $I\left(t\right)$. However, note that, if $\frac{I\left(t\right)}{S\left(t\right)}$ is unbounded as time tends to infinity then, $N\left(t\right)=S\left(t\right)+I\left(t\right)+R\left(t\right)$ is equivalent to $1+\frac{I\left(t\right)}{S\left(t\right)}=\frac{N\left(t\right)-R\left(t\right)}{S\left(t\right)}$. If $S\left(t\right)\to 0$ as t→∞, $I\left(t\right)\to 0$ as t→∞, $\frac{I\left(t\right)}{S\left(t\right)}\to +\infty$ as t→∞ and $\left(N\left(t\right)-R\left(t\right)\right)\to 0$ as t→∞, then by taking limits in $1+\frac{I\left(t\right)}{S\left(t\right)}=\frac{N\left(t\right)-R\left(t\right)}{S\left(t\right)}$, we get the contradiction +∞=0. Thus, $\underset{t\to \infty }{\lim\;\sup}\frac{I\left(t\right)}{S\left(t\right)}<+\infty$. Property (i) has been proved.

Property (ii) is a direct consequence of the constraints of the fact that the functions $f_{i}\left({\hat{c}}_{i}\;,\;t\right):R_{0+}\to R_{0+}$ are all continuous for any $i\in \bar{\chi }$, Property (i) and Equation (25), via (26), since $f\left(0\right)$ has a positive either finite or infinity value and each $f_{i}\left({\hat{c}}_{i}\;,t\right)$; $i\in \bar{\chi }$ is positively bounded at infinity as well. As a result, by defining a function that is a straight line $g\left(c\right)=c$ crossing zero of unity slope, one finds that g(0)=0<f(0) and for each given ti,ti+1∈Imp, there is a finite admissibility interval $\left[0,\;{\bar{c}}_{i}\right)$ for $c_{i}$, such that ${\bar{c}}_{i}={\bar{c}}_{i}\left(c_{1}\;,c_{2}\;,\ldots ,c_{i-1}\right)$; $i\in \bar{\chi }$, such that $c_{i}=g\left(c_{i}\right)<f_{i}\left({\hat{c}}_{i,}t\right)$; $i\in \bar{\chi }$ for all $t\in \left[t_{i}\;,\;t_{i+1}\right)$ since $f_{i}\left({\hat{c}}_{i,}t\right)$; $i\in \bar{\chi }$ is positively lower bounded. Property (ii) has been proved. □

**Proof** **of** **Theorem** **3.** By zeroing the time derivatives of (1) and (3) with $I_{df}=0$, one gets:

(A3) $$\left(1-q_{df}\right)A-\left(\mu +l_{df}\right)S_{df}+\rho R_{df}=q_{df}A+l_{df}S_{df}-\left(\mu +\rho \right)R_{df}=0$$

which leads to

(A4) $$R_{df}=\frac{q_{df}A+l_{df}\left(N_{df}-R_{df}\right)}{\mu +\rho }$$

Additionally, by summing-up (1)–(3) with the three derivatives being zero, one gets $0={\dot{N}}_{df}=-\mu N_{df}+A=-\mu \left(S_{df}+R_{df}\right)+A=0$ leading to

(A5) $$N_{df}=S_{df}+R_{df}=\frac{A}{\mu }$$

The replacement of (A5) into (A5) yields (27) which substituted into $S_{df}=\frac{A}{\mu }-R_{df}$ yields (28). □

**Proof** **of** **Theorem** **4.** It follows from (1) and (4) that the inclusion of all the potential vaccination controls in the susceptible subpopulation results in the following dynamics:

$$\dot{S}\left(t\right)=\left(1-q\left(t\right)\right)A+\rho R\left(t\right)-\left(\mu +\frac{\beta \left(t\right)I\left(t\right)}{1+\alpha_{1}S\left(t\right)+\alpha_{2}I\left(t\right)}+l\left(t\right)+k\left(t\right)\delta \left(0\right)\right)S\left(t\right)$$

for large enough initial conditions for the susceptible subpopulation $S_{0}\geq S_{df}+\varepsilon \left(0\right)$. For any $t\in R_{0+}$ if $\dot{S}\left(t\right)\leq 0$ then impulsive vaccination is not applied, i.e., $k\left(t\right)=0$. If $\dot{S}\left(t^{-}\right)>0$ then impulsive vaccination is applied at this time instant with gain $k\left(t\right)=1-\frac{S_{df}}{S\left(t^{-}\right)}-\varepsilon \left(t\right)$ with $0<\varepsilon \left(t\right)<1-\frac{S_{df}}{S\left(t^{-}\right)}$ so that $S\left(t\right)=\left(1-k\left(t\right)\right)S\left(t^{-}\right)<S\left(t^{-}\right)$ so that there is a finite jump down of the susceptible subpopulation at t∈Imp. Thus, one obtains that $\dot{S}\left(t\right)$ is minus infinity by the contribution to its value of $-k\left(t\right)\delta \left(0\right)S\left(t\right)$ since all of its contributing remaining right-hand side terms are finite. Furthermore, $\left(1-k\left(t\right)\right)S\left(t^{-}\right)-\varepsilon \left(t\right)S\left(t^{-}\right)=S_{df}$ and $S\left(t\right)=\left(1-k\left(t\right)\right)S\left(t^{-}\right)=S_{df}+\varepsilon \left(t\right)S\left(t^{-}\right)>S_{df}$. Note that $\dot{S}\left(t\right)<0$ for any $t\in \left[t_{i}\;,\;t_{i+1}\right)$ for any two consecutive ti ,ti+1∈Imp which implies that $S\left(t\right)$ is strictly decreasing on the interval $\left[t_{i}\;,\;t_{i+1}\right)$. Since $\dot{S}\left(t_{i}\right)$ is a minus infinity impulse, then $t_{i+1}-t_{i}$ has a nonzero finite measure and since εtiti∈Imp is strictly decreasing, there is a finite tχ∈Imp such that the amount $\left(S\left(t_{i}\right)-S_{df}\right)>0$ is small enough so that $\dot{S}\left(t\right)<0$ for some finite sufficiently small $\xi \in R_{+}$, such that t+ξ∉Imp, and $t\in \left(t_{\chi },t_{\chi }+\xi \right)$ with $\dot{S}\left(t_{\chi }+\xi \right)=0$ and $S\left(t_{\chi }+\xi \right)=S_{df}$ from the continuity of $S\left(t\right)$ on ${\left[t\;,\;t_{\chi }+\xi \right]}^{}$. □

**Proof** **of** **Theorem** **5.** If $I\left(t\right)<\frac{A-\mu N\left(t\right)}{\alpha }$ for any given $t\in R_{0+}$, then the differential equation $\dot{N}\left(t\right)=-\mu N\left(t\right)+A-\alpha I\left(t\right)$ implies that $\dot{N}\left(t\right)>0$. If $0<I_{0}<\frac{A-\mu \left(S_{0}+R_{0}\right)}{\alpha +\mu }$, it follows that $\dot{I}\left(t\right)<0$; $\forall t\in R_{0+}$ from Proposition 1 or Theorem 2 and then $I:R_{0+}\to R_{+}$ is strictly decreasing with $\underset{t\to \infty }{\lim}I\left(t\right)=\underset{t\to \infty }{\lim}\dot{I}\left(t\right)=0$. From Theorem 3 and an assumption in Theorem 4, $S_{0}>S_{df}$. Then, from Theorem 4, $\;S:R_{0+}\to R_{+}$ is strictly decreasing with $\dot{S}\left(t\right)<0$ if t∉Imp and $S\left(t\right)<S\left(t^{-}\right)$ if t∈Imp, Imp having a finite cardinal, and $S\left(t^{-}\right)\neq 0$, and $\underset{t\to \infty }{\lim}S\left(t\right)=S_{df};\;\underset{t\to \infty }{\lim}\dot{S}\left(t\right)=0$. Since $0<I_{0}<\frac{A-\mu \left(S_{0}+R_{0}\right)}{\alpha +\mu }$ and $I:R_{0+}\to R_{+}$ is strictly decreasing, then $I\left(t\right)\leq I_{0}<\frac{A-\mu \left(S_{0}+R_{0}\right)}{\alpha +\mu }$; $\forall t\in R_{0+}$, with the first inequality being equality if and only if t=0. As a result, $\dot{N}\left(t\right)>0$; $\forall t\in R_{0+}$ with existing limits $\underset{t\to \infty }{\lim}N\left(t\right)<+\infty$, $\underset{t\to \infty }{\lim}\dot{N}\left(t\right)=0$ so that $N:R_{0+}\to R_{+}$ is strictly increasing and bounded above with limit $N_{df}=A/\mu$ as time tends to infinity. Since $I,\;S:R_{0+}\to R_{+}$ are strictly decreasing, $R_{0}<R_{df}$ and $N:R_{0+}\to R_{+}$ then $R:R_{0+}\to R_{+}$ is strictly increasing with limit $R_{df}^{}$ as time tends to infinity. Property (i) has been proved. To prove Property (ii), assume that $I_{0}=\frac{A-\mu \left(S_{0}+R_{0}\right)}{\alpha +\mu }$ and $R_{0}>R_{df}$. From the assumptions of Theorem 4, $S_{0}>S_{df}$. Thus, one gets:

$$I_{0}=\frac{A-\mu \left(S_{0}+R_{0}\right)}{\alpha +\mu }<\frac{A-\mu \left(S_{df}+R_{df}\right)}{\alpha +\mu }=\frac{A-\mu N_{df}}{\alpha +\mu }=\frac{A-A}{\alpha +\mu }=0$$

which contradicts $I_{0}\geq 0$, so that $I_{0}>\frac{A-\mu \left(S_{0}+R_{0}\right)}{\alpha +\mu }$ if $I_{0}\geq \frac{A-\mu \left(S_{0}+R_{0}\right)}{\alpha +\mu }$ and $R_{0}>R_{df}$. Now, since $I_{0}>\frac{A-\mu \left(S_{0}+R_{0}\right)}{\alpha +\mu }$ and $R_{0}>R_{df}$ then, from Proposition 1/Theorem 2, and since the infectious subpopulation evolution is a strictly decreasing function, there exists a finite $t_{1}\in R_{+}$ such that

$$I\left(t_{1}\right)=\frac{A-\mu \left(S\left(t_{1}\right)+R\left(t_{1}\right)\right)}{\alpha +\mu }=\frac{A-\mu N_{df}}{\alpha +\mu }=\frac{A-A}{\alpha +\mu }=0$$

and $\dot{I}\left(t\right)<0$ and $\dot{N}\left(t\right)>0$; $\forall t\left(>t_{1}\right)\in R_{0+}$ so that $R\left(t\right)$ is increasing on $\left(t_{1}\;,\;\infty \right)$, since $R\left(t\right)\geq R\left(t^{-}\right)$ by the non-negative contribution of any impulsive vaccination (or $R\left(t\right)=R\left(t^{-}\right)$ in its absence); $\forall t\in R_{0+}$, so that the solution trajectory cannot converge to the disease-free equilibrium point according to Property (i) unless the convergence $R\left(t\right)\to R_{df}$ has taken place on $\left[0\;,\;t_{1}\right]$. Thus, one concludes that in order for the solution trajectory to converge asymptotically to the disease-free equilibrium point, it has to hold that either $R\left(t\right)\leq R_{df}$ for all $t\in R_{0+}$ and $R\left(t\right)\to R_{df}$ as t→∞, or $R_{0}>R_{df}$ and $R\left(t\right)=R_{df}$ with $\dot{R}\left(t\right)=0$ for $t\in \left[t_{1}-\varepsilon \;,\;\infty \right)$ and some $\varepsilon \in R_{0+}$ since $R:R_{0+}\to R_{+}$ is continuous. □

## References

[1] Kermack W.O., McKendrick A.G. (1927). A contribution to the mathematical theory of epidemics. Proc. R. Soc. Lond. Ser. A Math. Phys. Sci..

[2] Hethcote H.W., Lewis M., Driessche P.V.D. (1989). An epidemiological model with a delay and a nonlinear incidence rate. J. Math. Biol..

[3] Delamater P.L., Street E.J., Leslie T.F., Yang Y.Y., Jacobsen K.H. (2019). Complexity of the basic reproduction number. Emerg. Infect. Dis..

[4] Greenwood B. (2014). The contribution of vaccination to global health: Past, present and future. Philos. Trans. R. Soc. Biol. Sci..

[5] Etxeberria-Iriondo A., De la Sen M., Alonso-Quesada S. A new epidemic model under vaccination. Proceedings of the 2019 14th IEEE Conference on Industrial Electronics and Applications (ICIEA 2019).

[6] De la Sen M., Alonso-Quesada S. (2011). Vaccination strategies based on feedback control techniques for a general SEIR-epidemic model. Appl. Math. Comput..

[7] Nino I., Fernandez M., De la Sen M., Alonso-Quesada S., Nistal R. About two compared SEIARD and SEIR discrete epidemic models. Proceedings of the 17th International Conference on ICT and Knowledge Engineering (ICT &KE) (ICIEA 2019).

[8] De la Sen M., Alonso-Quesada S., Ibeas A. (2015). On the stability of an SEIR epidemic model with distributed time-delay and a general class of feedback vaccination rules. Appl. Math. Comput..

[9] Zhai S., Luo G., Huang T., Wang X., Tao J., Zhou P. (2021). Vaccination control of an epidemic model with time delay and its application to COVID-19. Nonlinear Dyn..

[10] Wintachai P., Prathom K. (2021). Stability analysis of SEIR model related to efficiency of vaccines for COVID-19 situation. Heliyon.

[11] Cai Y., Li J., Kang Y., Wang K., Wang W. (2020). The fluctuation impact of human mobility on the influenza transmission. J. Frankl. Inst. Eng. Appl. Math..

[12] Gui K., Ma W.B. (2020). Global dynamics of an SI epidemic model with nonlinear incidence rate, feedback controls and time delays. Math. Biosci. Eng..

[13] Qureshi S. (2020). Real life application of Caputo fractional derivative for measles epidemiological autonomous dynamical system. Chaos Solitons Fractals.

[14] Yang H.M., Freitas A.R.R. (2019). Biological view of vaccination described by mathematical modellings: From rubella to dengue vaccines. Math. Biosci. Eng..

[15] Cai Y., Kang Y., Banerjee M., Wang W. (2016). A stochastic epidemic model incorporating media coverage. Commun. Math. Sci..

[16] De la Sen M., Agarwal R.P., Ibeas A., Alonso-Quesada S. (2010). On a generalized time-varying SEIR epidemic model with mixed point and distributed time-varying delays and combined regular and impulsive vaccination controls. Adv. Differ. Equ..

[17] De la Sen M., Agarwal R.P., Ibeas A., Alonso-Quesada S. (2011). On the existence of equilibrium points, boundedness, oscillating behavior and positivity of a SVEIRS epidemic model under constant and impulsive vaccination. Adv. Differ. Equ..

[18] Darti I., Suryanto A. (2020). Dynamics of a SIR epidemic model of childhood diseases with a saturated incidence rate: Continuous model and its nonstandard finite difference discretization. Mathematics.

[19] Wang J. (2021). Dynamics and bifurcation analysis of a state-dependent impulsive SIS model. Adv. Differ. Equ..

[20] Duro A., Piccione V., Ragusa M.A., Veneziano V. (2014). New enviromentally sensitive patch index-ESPI-for MEDALUS protocol. AIP Conference Proceedings 2014.

[21] Bansal S., Grenfell B.T., Meyers L.A. (2007). When individual behaviour matters: Homogeneous and network models in epidemiology. J. R. Soc. Interface.

[22] Long Y., Wang L. (2019). Global dynamics of a delayed two-patch discrete SIR disease model. Commun. Nonlinear Sci. Numer. Simul..

[23] De La Sen M., Ibeas A., Alonso-Quesada S., Nistal R. (2019). On a SIR Model in a Patchy Environment Under Constant and Feedback Decentralized Controls with Asymmetric Parameterizations. Symmetry.

[24] Wankudu D. (2017). Complete global analysis of a two-scale network SIRS epidemic dynamic model with distributed delay and random perturbations. Appl. Math. Comput..

[25] Li H., Peng R. (2019). Dynamics and asymptotic profiles of endemic equilibrium for SIS epidemic patch models. J. Math. Biol..

